# Electrocatalytic CO_2_ Reduction over Cu_3_P Nanoparticles Generated via a Molecular Precursor Route

**DOI:** 10.1021/acsaem.0c01360

**Published:** 2020-10-27

**Authors:** Courtney A. Downes, Nicole J. Libretto, Anne E. Harman-Ware, Renee M. Happs, Daniel A. Ruddy, Frederick G. Baddour, Jack R. Ferrell III, Susan E. Habas, Joshua A. Schaidle

**Affiliations:** † Catalytic Carbon Transformation and Scale-Up Center, National Renewable Energy Laboratory, 15013 Denver West Parkway, Golden, Colorado 80401, United States; ‡ Davidson School of Chemical Engineering, Purdue University, West Lafayette, Indiana 47907, United States; § Renewable Resources and Enabling Sciences Center, National Renewable Energy Laboratory, 15013 Denver West Parkway, Golden, Colorado 80401, United States

**Keywords:** metal phosphide nanoparticles, copper phosphide, electrocatalysis, carbon utilization, CO_2_ reduction

## Abstract

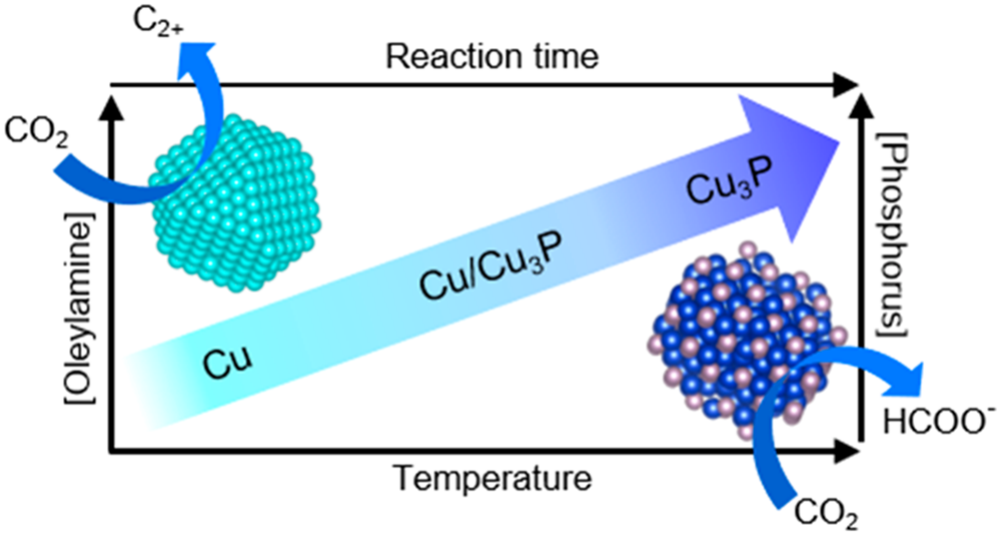

The design of nanoparticles (NPs) with tailored morphologies and finely tuned electronic and physical properties has become a key strategy for controlling selectivity and improving conversion efficiency in a variety of important electrocatalytic transformations. Transition metal phosphide NPs, in particular, have emerged as a versatile class of catalytic materials due to their multifunctional active sites and composition- and phase-dependent properties. Access to targeted transition metal phosphide NPs with controlled features is necessary to tune the catalytic activity. To this end, we have established a solution-synthesis route utilizing a molecular precursor containing M–P bonds to generate solid metal phosphide NPs with controlled stoichiometry and morphology. We expand here the application of molecular precursors in metal phosphide NP synthesis to include the preparation of phase-pure Cu_3_P NPs from the thermal decomposition of [Cu(H)(PPh_3_)]_6_. The mechanism of [Cu(H)(PPh_3_)]_6_ decomposition and subsequent formation of Cu_3_P was investigated through modification of the reaction parameters. Identification and optimization of the critical reaction parameters (i.e., time, temperature, and oleylamine concentration) enabled the synthesis of phase-pure 9–11 nm Cu_3_P NPs. To probe the multifunctionality of this materials system, Cu_3_P NPs were investigated as an electrocatalyst for CO_2_ reduction. At low overpotential (−0.30 V versus RHE) in 0.1 M KHCO_3_ electrolyte, Cu_3_P-modified carbon paper electrodes produced formate (HCOO−) at a maximum Faradaic efficiency of 8%.

## Introduction

The projected availability of abundant renewable electrons presents an opportunity to sustainably produce valuable chemical and fuel products from waste streams and renewable carbon sources such as CO_2_ and biomass.^1−4^ These underutilized feedstocks can serve as not only precursors for the synthesis of fuels and chemicals but also liquid energy carriers to improve power grid management by mitigating the impact of the intermittency associated with renewable energy generation. However, the marriage of these renewable electrons with underutilized renewable feedstocks to improve grid stability and displace fossil-derived chemical production requires the development of economical routes for the electrochemical transformations with high selectivity and Faradaic efficiency (FE). These critically important transformations are challenging because of the multiple proton and electron transfers and bond breaking and bond forming steps needed to generate valuable products.^1−3^ Additionally, undesirable reactions such as H_2_ evolution are thermodynamically competitive under the reaction conditions utilized for these transformations, which can lead to reduced efficiencies toward valuable products. Therefore, the development of catalytic systems to suppress competitive reactions is necessary to improve the overall energy efficiency and viability of the electrochemical conversion of waste streams and renewable carbon sources to valuable fuels and chemicals.

Electrocatalysts with sophisticated features, such as multifunctional active sites and tailored electronic properties, have been explored to overcome the difficulties associated with these transformations.^5−8^ Transition metal phosphides have emerged as a versatile materials platform with interesting structural and electronic properties that have led to exemplary catalytic performance for important energy conversions^9,10^ such as electrocatalytic hydrogen evolution.^11,12^ The incorporation of phosphorus into the metal structure imparts unique electronic properties, such as extensive hybridization of the M and P orbitals and charge transfer between the M and P sites.^13^ Surface phosphorus sites can also actively participate in the catalytic transformation through direct coordination with or stabilization of reaction intermediates.^11,14^ Because of this colocalized multifunctionality, tuning the stoichiometry and corresponding phase of metal phosphides confers a high degree of control over the catalytic selectivity, efficiency, and activity.

Despite the interesting properties of transition metal phosphides, the favorable thermodynamics and kinetics of H_2_ evolution in aqueous solution has traditionally limited their application as electrocatalysts for other important energy conversions such as the electrochemical reduction of CO_2_.^15^ However, there are several recent examples of transition metal phosphides electrochemically reducing CO_2_ selectively to C_1_ and even more complex C_3_ and C_4_ oxygenates.^16−18^ Bulk nickel phosphides (Ni_3_P, Ni_2_P, Ni_12_P_5_, Ni_5_P_4_, and NiP_2_) were observed to electrochemically reduce CO_2_ to methylglyoxal and 2,3-furandiol via formate as an intermediate reaching a maximum FE of 71% for 2,3-furandiol at 0.00 V versus RHE over Ni_2_P.^16^ Higher conversion of CO_2_ to C_3_ and C_4_ oxygenates, rather than formate and H_2_, was observed for the more phosphorus-rich nickel phosphides, implicating phosphorus in the enhancement of CO_2_ reduction efficiency through the formation of reactive hydrides and nucleophilic CO_2_ binding sites that facilitate the generation of formate and C_3+_ oxygenates. The phosphorus-dependent CO_2_ reduction activity observed for nickel phosphides highlights the advantage of colocalized multifunctionality.^19^ A Cu_3_P/C composite was also shown to electrochemically reduce CO_2_ to CO at −0.30 V versus RHE with an average FE of 47%.^18^ These results suggest the possibility of transition metal phosphides serving as a unique materials platform whereby CO_2_ reduction can outperform H_2_ evolution within a certain potential range.

Due to the unique CO_2_ reduction capabilities of copper-based electrocatalysts^20−22^ and the copper-based phosphide material,^18^ we identified nanostructured Cu_3_P as a promising synthetic target. Nanostructuring offers the benefits of high surface area and efficient metal utilization, and provides a tunable platform whereby the structural, physical, and electronic properties can be controlled to achieve the desired catalytic activity.^5,7^ Solution-based synthesis methods have emerged as a viable strategy to produce nanostructured transition metal phosphides;^13,14^ however, solution synthesis routes to Cu_3_P nanoparticles (NPs) are limited^23−27^ in comparison to other metal phosphides. A solution synthesis route to NP nickel, rhodium, and palladium phosphides (Ni_2_P, Rh_2_P, and Pd_3_P) was recently reported using commercially available molecular precursors, which afforded control over the morphology and phase of the resultant metal phosphide NPs.^28^ The metal phosphide NPs synthesized from the molecular precursor route were shown to catalyze a variety of thermochemical and electrochemical transformations.^28−30^ Herein, we present a synthetic route to phase-pure Cu_3_P NPs via thermal decomposition of the molecular precursor [Cu(H)(PPh_3_)]_6_ and elucidate the mechanism of NP formation through an investigation of key reaction parameters. The resulting Cu_3_P NPs were evaluated as electrocatalysts for CO_2_ reduction in CO_2_-saturated KHCO_3_ aqueous solutions.

## Experimental Section

### General

Synthetic manipulations to prepare Cu_3_P NPs were conducted in a N_2_ atmosphere using standard Schlenk techniques or in a N_2_-filled Vacuum Atmospheres glovebox. Oleylamine (OAm, 70% technical grade) and 1-octadecene (ODE, 90%) were purchased from Sigma-Aldrich and dried prior to use by heating to 120 and 150 °C, respectively, under vacuum for 5 h and stored in a N_2_-filled glovebox. Triphenylphosphine (PPh_3_, 99%) was purchased from Sigma-Aldrich, and [Cu(H)(PPh_3_)]_6_ (96%) was purchased from Acros Organics and stored in a N_2_-filled glovebox. Water used for electrochemistry experiments was purified by a Milli-Q Water Purification System with a specific resistance of 18.2 MΩ·cm at 25 °C. Standards for high-performance liquid chromatography (HPLC) experiments were purchased from AccuStandards. Deuterium oxide (D 99.9%) containing 0.05 wt % 3-(trimethylsilyl)propionic-2,2,3,3-*d*_4_ acid (Na salt), carbon tetrachloride (99.99% ACS reagent), tetrachloroethylene, potassium bicarbonate (99.95%), sodium chloride (99.999%), and Chelex 100 (Na form) were purchased from Sigma-Aldrich. Fluorine doped tin oxide (FTO) coated glass was purchased from Techinstro.

### Synthesis of Cu_3_P Nanoparticles

In a three-neck round-bottom flask fitted with a condenser and two septa, [Cu(H)(PPh_3_)]_6_ (0.326 g, 1 mmol Cu) was combined with dried OAm (4.9 mL, 15 mmol) and ODE (8.0 mL, 20 mmol) and heated to 250 °C under N_2_ with rapid stirring. The mixture was held at 250 °C for 30 min and then heated to 320 °C (ca. 3.5 °C/min). The reaction was maintained at 320 °C for 15 min, followed by the removal of the heat source and ambient cooling. Approximately 5 mL of CHCl_3_ was added to the reaction mixture in air followed by the addition of 30 mL of ethanol to flocculate the particles, which were then separated by centrifugation at 10000 rpm for 5 min. This washing procedure was performed an additional 4 times to remove impurities and OAm was added (ca. 0.5–1.0 mL) to each wash to ensure retention of the dispersibility of the NPs. The washed NPs were dried and stored in a N_2_-filled glovebox prior to evaluation. Following the complete washing procedure, a 60% yield of Cu_3_P NPs was calculated from elemental analysis of the Cu concentration of a representative sample. Cu_3_P NPs were supported on carbon powder by adding a CHCl_3_ suspension of NPs dropwise to a rapidly stirring suspension of Vulcan XC 72R (Cabot) in CHCl_3_ (ca. 100 mL). The mixture was sonicated for 5 min, stirred overnight, and recovered by centrifugation at 8000 rpm for 10 min. The resultant carbon-supported Cu_3_P NPs were dried under vacuum overnight and stored in an N_2_-filled glovebox.

### Modification of Cu_3_P Reaction Parameters

To understand the mechanism of Cu_3_P formation, the following reaction parameters were modified: time, temperature, OAm concentration, and phosphorus concentration. A 30 min hold at 250 °C was employed for all reactions. Following the hold at 250 °C, the reaction mixture was heated to the target temperature and held for the designated time. To assess the role of phosphorus concentration, PPh_3_ (2–4 equiv relative to Cu) was added to the reaction mixture containing [Cu(H)(PPh_3_)]_6_, OAm (15 mmol), and ODE (25 mmol). Cu_3_P NPs synthesized in the presence of added PPh_3_ are denoted generally as P–Cu_3_P with OAm–P–Cu_3_P and R–P–Cu_3_P referring to the as-synthesized and reduced derivatives, respectively. When the OAm concentration was changed, ODE was used to keep the total solvent volume at 12.9 mL. Aliquots (0.5 mL) of the reaction mixture were extracted via syringe over the duration of the experiment to investigate the properties of the NPs at specific temperatures and times. The aliquots were purified by addition of ethanol followed by centrifugation to separate the particles.

### Characterization of Cu_3_P

Powder X-ray diffraction (XRD) data on the Cu_3_P materials were collected using a Rigaku Ultima IV diffractometer with a Cu Kα source (40 kV, 44 mA). Diffraction patterns were collected in the 2θ range of 20–80° at a scan rate of 4°/min. The as-prepared NPs were drop-cast onto a glass slide from a chloroform suspension. The resulting patterns were compared to powder diffraction files (PDF) from the International Centre for Diffraction Data (ICDD). NIST Si standard was used to calibrate the XRD peak positions. For transmission electron microscopy (TEM) analysis, the unsupported NPs were drop-cast onto continuous carbon-coated copper grids (Ted Pella part no. 01824) from chloroform suspensions. Imaging was performed using an FEI G^2^ T20 Tecnai TEM operated at 200 kV and an FEI Tecnai G^2^ ST30 TEM operated at 300 kV, and all image analysis was conducted with ImageJ software.^31^ Lattice spacings were measured from the fast-Fourier transforms (FFTs) of high-resolution TEM (HRTEM) images. Size distributions were determined from a manual diameter measurement of >100 particles. The metal and phosphorus loadings of the as-prepared NPs and the reaction yield were determined by inductively coupled plasma optical emission spectroscopy (ICP-OES) performed by Galbraith Laboratories Inc., (Knoxville, TN). For FTIR spectroscopy analysis, the as-prepared NPs were drop-cast onto a silicon wafer and analyzed using a Thermo Scientific Nicolet 6700 FT-IR Spectrometer (4000 to 1500 cm^–1^, 32 scans, 4 cm^–1^ resolution). UV–vis-NIR spectroscopy analysis of as-prepared Cu_3_P NPs suspended in CCl_4_ and C_2_Cl_4_ were conducted using an Agilent Technologies Cary Series UV–vis-NIR Spectrophotometer (2500 to 500 nm, 600 nm/min). *In situ* X-ray absorption spectroscopy (XAS) measurements were performed at the 10-BM beamline at the Advanced Photon Source (APS) at Argonne National Laboratory. All measurements were performed at the Cu K edge (8.979 keV) in transmission mode in fast scan from 250 eV below the edge to 800 eV above the edge, which took approximately 10 min per scan. At the Cu K edge, the Cu–P (CN = 1, R = 2.34 Å), Cu–Cu (CN = 1, R = 2.56 Å), and Cu–O (CN = 1, R = 1.95 Å) scattering pairs were simulated. S_o_^2^ was calibrated by fitting the Cu foil, which gave a value of 0.67. A least squared fit the first shell of r-space and isolated q-space were performed on the k^2^ weighted Fourier transform data over the range 2.7 to 11 Å^–1^ in each spectrum to fit the magnitude and imaginary components. XAS measurements were performed on Cu_3_P NPs synthesized in the presence of 2 equiv of PPh_3_ that were supported on carbon to achieve a nominal loading of 5 wt % (P–Cu_3_P/C). P–Cu_3_P/C was pressed into a stainless-steel sample holder and placed in a sample cell. The cell was sealed and transferred to the beamline for measurement. XAS measurements were first collected on the as-synthesized carbon-supported Cu_3_P NPs (OAm–P–Cu_3_P/C) at ambient temperature. OAm–P–Cu_3_P/C was then treated in flowing 5% H_2_/He (100 sccm) at 450 °C for 2 h to generate reduced carbon-supported Cu_3_P NPs (R–P–Cu_3_P/C), cooled to ambient temperature in He (100 sccm), sealed, and transferred to the beamline for a second measurement. Without exposure to air, R–P–Cu_3_P/C was then passivated by treating in flowing 1% O_2_/He (100 sccm) at ambient temperature for 1 h, sealed in 1% O_2_, and transferred to the beamline for a third measurement.

### Preparation of Cu_3_P-Modified Working Electrodes

Coiled copper wire was affixed to carbon paper (CP) (Fuel Cell Store, Spectracarb 2050A 0850) using silver epoxy and allowed to dry. The copper wire was fitted through a glass tube and two-part epoxy was used to cover the exposed copper wire and silver epoxy, and to define a 1 cm × 1 cm working area. Once the epoxy was dried, as-synthesized Cu_3_P-modified CP (OAm–Cu_3_P/CP) were prepared by drop-casting Cu_3_P NPs suspended in chloroform onto CP to produce loadings of 1–1.5 mg/cm^2^. The loadings were calculated based on the total mass of NPs. As-synthesized Cu_3_P-modified FTO electrodes were prepared in an analogous fashion to OAm–Cu_3_P/CP. To prepare reduced Cu_3_P-modified CP electrodes (R–Cu_3_P/CP), Cu_3_P NPs were suspended in chloroform and drop cast onto CP to produce loadings of 1 mg/cm^2^. After drying, the Cu_3_P-modified carbon papers were reduced in a tube furnace in flowing (500 sccm) 5% H_2_/N_2_. The temperature was increased at 5 °C/min to 450 °C and held for 2 h. R–Cu_3_P/CP were cooled to below 50 °C in flowing 5% H_2_/N_2_. Due to the air-sensitive nature of Cu_3_P and the inability to completely eliminate air exposure when preparing and executing the electrochemistry experiments, the samples were passivated in flowing (500 sccm) 1% O_2_/N_2_ for 1 h at ambient temperature. This passivation step allows for a controlled oxidation of the catalyst surface in contrast to the rapid oxidation expected upon directly exposing the reduced catalyst to air. Coiled copper wire was affixed to R–Cu_3_P/CP using silver epoxy and allowed to dry. The copper wire was fitted through a glass tube, and two-part epoxy was used to cover the exposed copper wire and silver epoxy and to define a 1 cm × 1 cm working area. The Cu_3_P-modified working electrodes were stored in a N_2_-filled glovebox prior to use.

### Electrochemical Methods

Electrochemical measurements were performed in 0.1 and 0.5 M KHCO_3_ aqueous electrolyte in a two-compartment, three-electrode cell utilizing a Metrohm Autolab potentiostat. Metallic impurities in the as-prepared electrolyte were removed before electrolysis by chelating the solution with Chelex 100. Potassium bicarbonate electrolytes were sparged with CO_2_ for 30 min prior to electrochemical analysis. The two compartments were separated by an anion-exchange membrane (Selemion AMV AGC Inc.). The working chamber of the cell was continuously purged with CO_2_. A graphite rod was used as the counter electrode and the reference electrode was an Ag/AgCl (3 M NaCl) electrode separated from the cell by a Vycor frit. Electrochemical impedance spectroscopy was employed to measure the uncompensated resistance (R_u_) of the electrochemical cell. The potentiostat’s IR compensation function was used to compensate 85% R_u_. The final 15% of R_u_ was mathematically corrected for after the electrochemical data was collected.

### CO_2_ Reduction Product Analysis

Gas phase products were quantified with gas chromatography (Agilent Technologies 7890A), equipped with a thermal conductivity detector and a flame ionization detector. The catholyte and anolyte from each electrolysis experiment were analyzed by nuclear magnetic resonance (NMR) spectroscopy to quantify liquid products, and the NMR results from select electrolysis experiments were confirmed by high-performance liquid chromatography (HPLC). A Bruker Avance Nanobay spectrometer at 9.4 T (400 MHz) equipped with a Bruker 5 mm BBO probe was used to collect ^1^H spectra of the electrolyte samples. Both catholyte and anolyte samples were analyzed for liquid products to account for product crossover. Suppression of the water peak was achieved using WATERGATE,^32^ a recycle delay of 1 s, and a total of 64 scans. Pulse calibration was performed on each electrolyte sample prior to beginning the NMR spectroscopy experiment to account for the KHCO_3_ concentration differences between samples. Electrolyte samples were added to an NMR tube containing a capillary filled with 0.05 wt % TSP in D_2_O, which was used as the internal standard and reference for the NMR spectroscopy experiments. A calibration curve for formate in KHCO_3_ solutions was generated for product quantification. HPLC analysis of the catholyte and anolyte samples was performed using Agilent 1260 LC with a refractive index detector (RID) and 0.02 N H_2_SO_4_ mobile phase. Samples were prepared using neat reaction media (0.1 or 0.5 M KHCO_3_) and adjusted to pH 4 using H_2_SO_4_. An HPX-87H Aminex column was used with 20 uL injection volume with 0.5 mL/min mobile phase flow rate for 19 min, followed by 0.6 mL/min until 35 min, and final flow rate of 0.5 mL for 15 min, giving a method run time of 50 min. The RID was 55 °C, and the column temperature was programmed at 30 °C. The concentrations of analytes in standards were calculated relative to external calibrations using various standard solutions. The standards consisted of mix 1: formaldehyde and acetaldehyde (catalogue number M-8315); mix 2: hydroxyacetone, methanol, ethanol, acetone and 1-propanol (custom order); mix 3: glyoxal, methylgyoxal, propanal (custom order); and mix 4: formic acid, acetic acid, propionic acid, isobutyric acid, butyric acid, isovaleric acid, valeric acid, isocaproic acid, caproic acid, and heptanoic acid (catalogue number FAMQ-004) in water ranging in stock concentrations of 0.5–1.3 mg/mL. Stock standards were further diluted in 0.5 M KHCO_3_ previously adjusted to pH = 4 using H_2_SO_4_ to generate calibration concentrations ranging from 0.01 to 1 mg/mL.

## Results and Discussion

### Synthesis and Characterization of Cu_3_P Nanoparticles

The commercially available copper-phosphine hexamer, [Cu(H)(PPh_3_)]_6_, was identified as an attractive molecular precursor for the preparation of phase-pure Cu_3_P NPs because of the low valence state of copper and the presence of preformed Cu–P bonds. Thermal decomposition of [Cu(H)(PPh_3_)]_6_ (1 mmol Cu) in 15 mmol OAm and 25 mmol ODE was performed at 250 °C for 30 min to promote uniform decomposition of the precursor and homogeneous particle growth. The further heating of the reaction mixture to 320 °C and holding at this temperature for 15 min yielded phase-pure Cu_3_P NPs. The resultant NPs were extensively washed with ethanol and isolated in 60% yield. The ethanol wash was necessary to remove soluble impurities such as clusters that are commonly formed from polyhydrido-copper complexes like [Cu(H)(PPh_3_)]_6_.^33^ Without the extensive washing procedure with ethanol, impurities were observed by XRD. In comparison to the reported methods to access phase-pure Cu_3_P NPs, the procedure established here has the combined advantages of being a single-pot synthesis that requires only a short-reaction time (<1 h) and does not rely on highly reactive phosphine sources. The size and morphology of Cu_3_P NPs isolated by the synthetic procedure developed here are similar to those previously reported.

**Figure 1:**
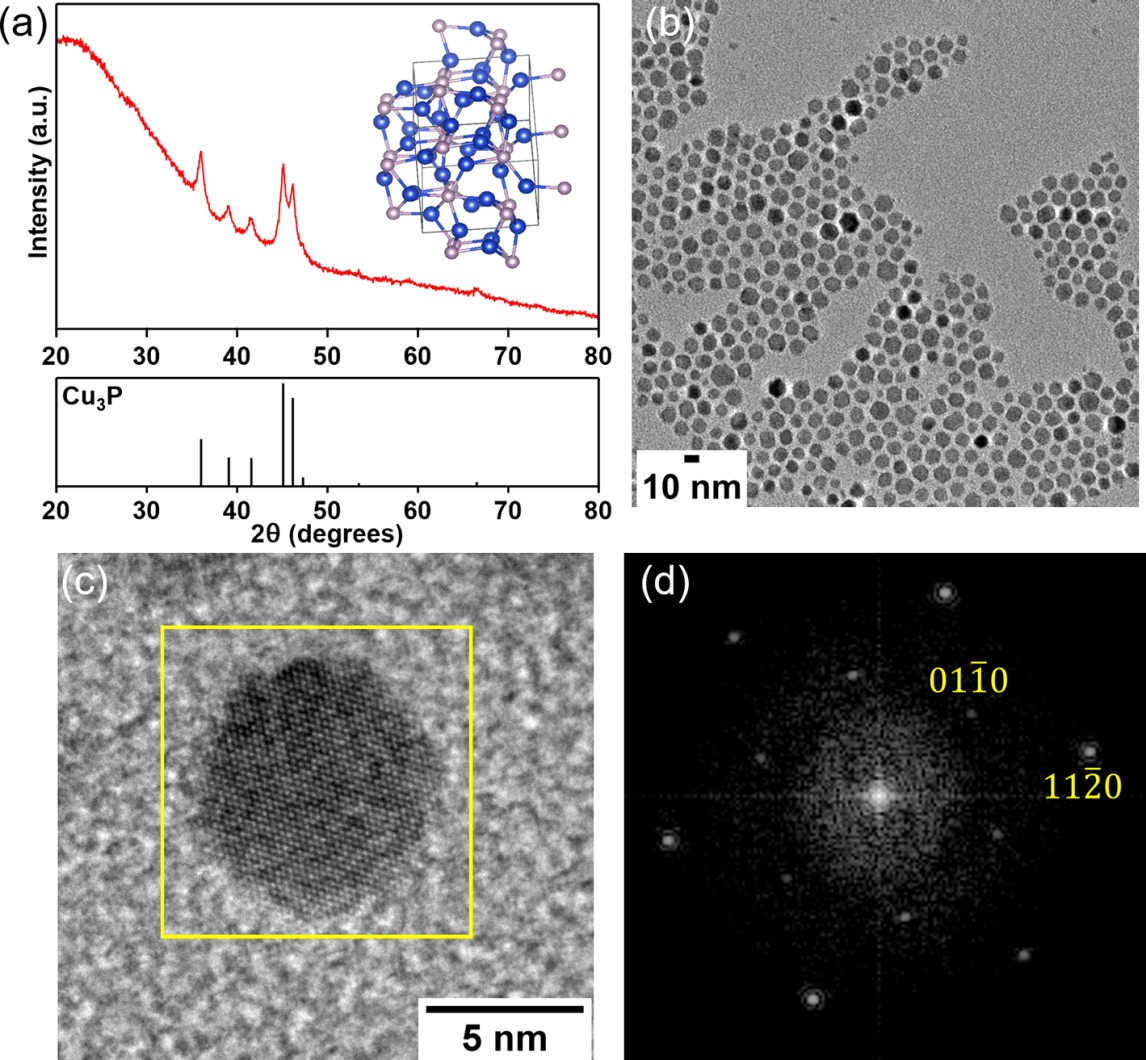
(a) XRD pattern of Cu_3_P NPs from 15 min reaction at 320 °C with corresponding crystal structure model (Cu: blue; P: pink) and PDF 01–071–2261 reference pattern (P6_3_*cm*), below. (b) TEM image of the Cu_3_P NPs, (c) HRTEM image with outlined region used for fast Fourier transform (FFT) analysis, and (d) FFT pattern indexed to the [0001] zone axis.

Analysis by XRD indicated the formation of hexagonal Cu_3_P (P6_3_*cm*) with good agreement to the reference pattern (PDF 01–071–2261) without any other observed crystalline phases, as shown in Figure 1a. The Cu_3_P NPs are single-crystalline and solid with a size distribution of 8.7 ± 1.6 nm as determined by analysis of TEM images (Figure 1b, Figure S1a). The high-resolution TEM (HRTEM) image of hexagonal Cu_3_P presented in Figure 1c and the corresponding FFT (Figure 1d) indexed to the [0001] zone axis have measured lattice spacings of 0.21 and 0.36 nm corresponding to the (112̅0) and (011̅0) crystal planes, respectively. Increasing the hold time at 320 °C to 30 min led to further growth of the NPs with analysis of TEM images indicating a size distribution of 10.3 ± 2.3 nm (Figure S1b). Longer reaction times of 1 h or more at 320 °C resulted in precipitation of nondispersible Cu_3_P aggregates. The optical properties of Cu_3_P were probed using UV–vis-NIR spectroscopy, and a plasmonic absorbance in the NIR region was observed (Figure S2). This plasmonic absorbance has been attributed to Cu vacancies in the NPs, and the presence and prevalence of these vacancies in copper phosphide NPs has led to the use of the formula Cu_3-x_P.^25,27,34^

### Effect of Modulating Reaction Conditions on Cu_3_P Formation

**Scheme 1:**
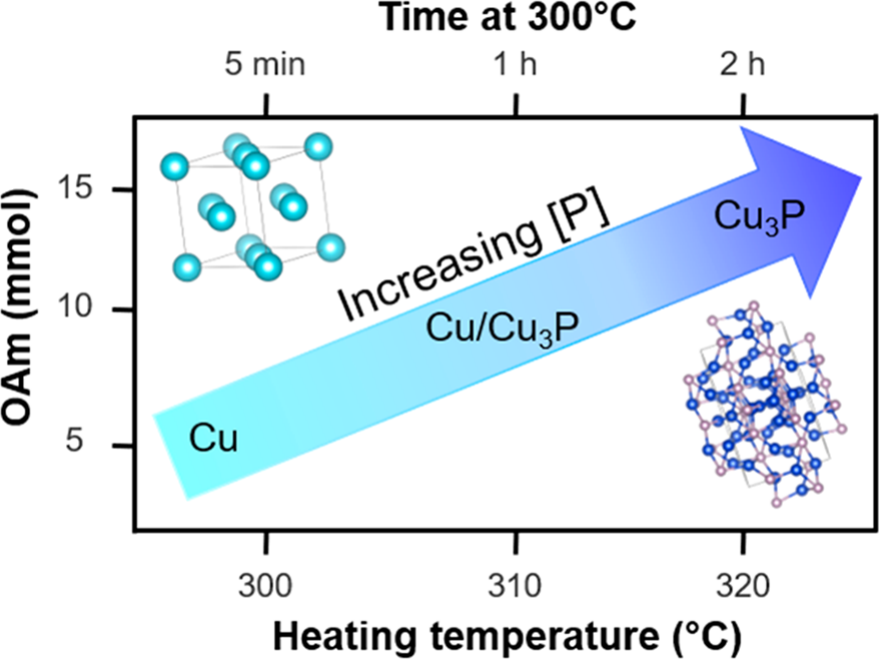
Mechanism of Cu_3_P Formation As a Function of Reaction Temperature, Time, and Oleylamine Concentration

Previous reports on the synthesis of Cu_3_P NPs suggested two primary mechanisms of NP formation: (1) generation of metallic Cu NPs followed by phosphidation or (2) direct, homogeneous nucleation of small Cu_3_P nuclei.^23−27^ These synthetic methods employed copper chloride as the Cu precursor and trioctylphosphine (TOP), triphenyl phosphite, phosphine gas, or tris(trimethylsilyl)phosphine as the phosphorus source. We sought to understand the mechanism of Cu_3_P formation when a single-source precursor that contains preformed Cu–P bonds was used in the absence of an external phosphorus source. To this end, a parametric exploration of the key reaction variables (i.e., time, temperature, and oleylamine concentration) was conducted to understand the transformation of [Cu(H)(PPh_3_)]_6_ into Cu_3_P NPs (Scheme 1).

#### Influence of Reaction Temperature and Time

The reaction conditions established for the synthesis of phase pure Cu_3_P involved heating [Cu(H)(PPh_3_)]_6_ in the presence of 15 mmol OAm and 25 mmol ODE at 250 °C for 30 min, followed by heating at 320 °C for 15 min. Although Cu_3_P NPs were also formed by directly heating [Cu(H)(PPh_3_)]_6_ to 320 °C without the 30 min hold at 250 °C, we employed this intermediate heating step to promote decomposition of [Cu(H)(PPh_3_)]_6_ and homogeneous formation of Cu_3_P from the NP precursors.^28^ XRD analysis of an aliquot of the reaction mixture removed after heating for 30 min at 250 °C indicated the presence of crystalline face-centered cubic Cu metal (Figure S3), revealing that Cu_3_P formation proceeded through a Cu intermediate (Scheme 1). TEM analysis of aliquots removed after 30 min at 250 °C and immediately upon reaching 280 °C revealed particles with average size distributions of 5.0 ± 1.4 nm and 4.8 ± 1.3 nm, respectively (Figure S4a,b). The resultant particles were prone to oxidation upon air exposure, as evidenced by conversion of the brown suspension to a green solid. To further understand the conversion of Cu to Cu_3_P, reaction mixtures were heated to a final temperature of 300 °C, 310 °C, and 315 °C and held at those temperatures for 30 min. This resulted in the formation of Cu, Cu/Cu_3_P, and Cu_3_P NPs (Figure S5a), respectively, indicating that increasing the temperature enhanced P-incorporation (Scheme 1).

**Figure 2:**
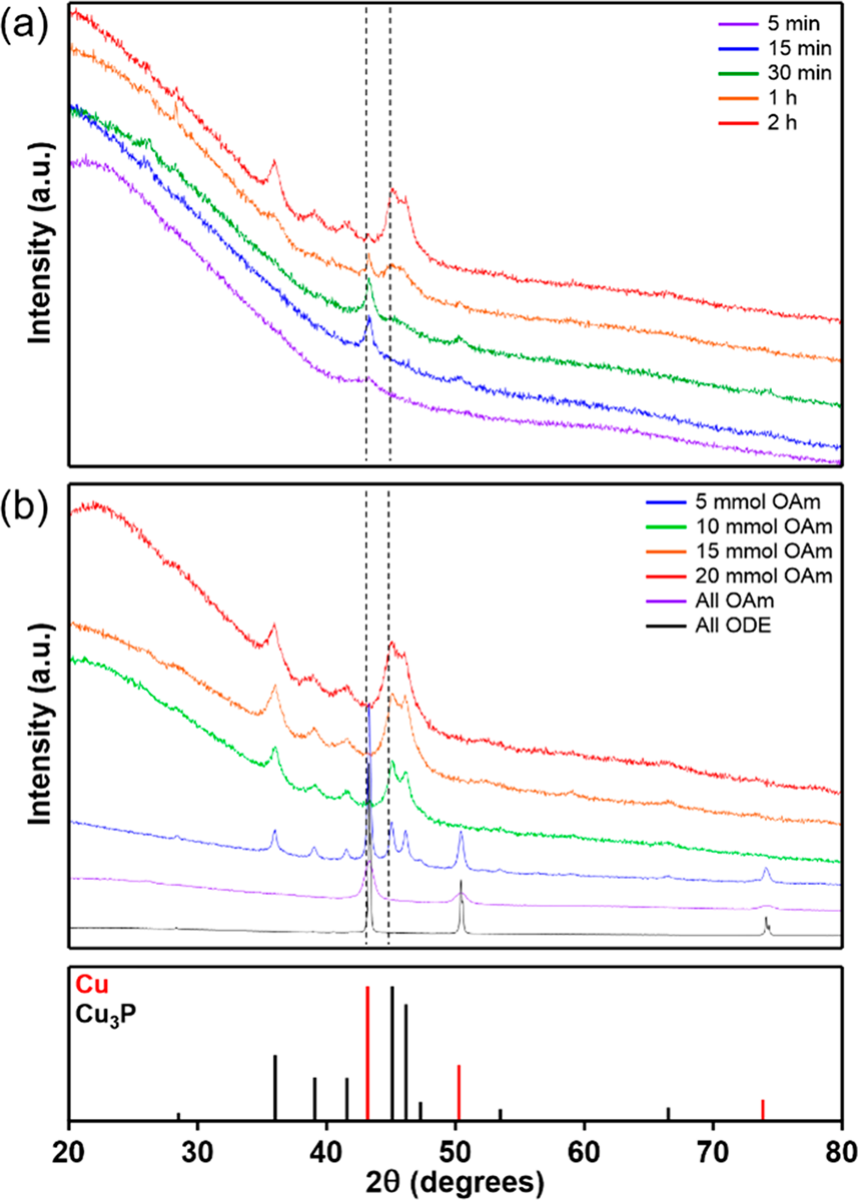
(a) XRD patterns after 5 min, 15 min, 30 min, 1 h, and 2 h reaction at 300 °C with an oleylamine concentration of 15 mmol. (b) XRD patterns of reaction products formed after 15 min at 320 °C with varying concentrations of OAm. Reference patterns for Cu and Cu_3_P are shown below, and the dotted lines on the experimental patterns indicate the highest intensity peak for Cu and Cu_3_P.

To explore how phosphorus incorporation changed with reaction time, the reaction mixture was heated at 300 °C with reaction times varied from 5 min to 2 h (Figure 2a). Cu NPs were readily formed at 300 °C after only 5 min with an average size distribution of 6.3 ± 1.2 nm (Figure S4c); however, the resultant particles rapidly turned green upon air exposure, indicating the formation of oxidized Cu species. As the reaction time was increased to 1 h, a broad peak in the XRD pattern between 45° and 50° emerged that is assigned to the formation of Cu_3_P (Figure 2a), and the particle size determined by TEM analysis increased to 7.2 ± 1.6 nm (Figure S 4d). The major Cu peak at 43° continued to diminish over 2 h as Cu was converted to Cu_3_P (Scheme 1) and this behavior was also observed at 310 °C (Figure S5b). These experiments reveal that phosphorus incorporation increases with reaction time and temperature and the transformation of [Cu(H)(PPh_3_)]_6_ to Cu_3_P proceeds through a Cu intermediate. It has been established that Ni NPs synthesized in the presence of alkylphosphines are phosphorus doped.^35,36^ Due to this literature precedent, the Cu intermediate generated here could contain phosphorus. Although we do not have conclusive evidence of phosphorus doping, the major XRD peak for the Cu synthesized from [Cu(H)(PPh_3_)]_6_ is shifted to higher 2θ (ca. 0.11 degrees) in comparison to the Cu reference, which could suggest a lattice contraction upon introduction of the smaller P into the Cu structure (Figure S6). Further work is needed to assess the viability of a P-doped Cu intermediate.

#### Influence of Oleylamine Concentration

The impact of OAm concentration on Cu_3_P formation was investigated by heating the reaction mixture at 320 °C for 15 min and varying the concentration of OAm while maintaining a constant reaction volume (12.9 mL total with balance of ODE) (Figure 2b). In the presence of only OAm or ODE, [Cu(H)(PPh_3_)]_6_ decomposed to metallic Cu, which precipitated rapidly from solution. Varying the OAm concentration from 5–20 mmol revealed that a balanced mixture of OAm (15–20 mmol) and ODE (20–25 mmol) was necessary to promote phosphidation and formation of phase-pure Cu_3_P (Figure 2b). TEM image analysis revealed the average particle size distribution increased from 8.7 ± 1.6 nm to 9.3 ± 3.3 nm (Figure S7) when the amount of OAm was increased from 15 to 20 mmol OAm. When 5 and 10 mmol of OAm was used, a mixture of Cu/Cu_3_P and Cu_3_P with trace Cu was produced, respectively (Figure 2b, Scheme 1). We also sought to expand our understanding of the role of OAm concentration on the rate of phosphorus incorporation at lower temperatures. To this end, 1–2 h reactions at 300 °C using 15 and 20 mmol of OAm were performed and the ratio of Cu to Cu_3_P was evaluated. At the higher OAm concentration, significant Cu metal was observed even after 2 h (Figure S8), indicating that high OAm concentrations reduce the phosphidation rate. Investigation into the formation of nickel phosphide NPs has also identified excess OAm as hindering phosphorus incorporation and favoring metal-rich phases.^37,38^

#### Influence of Phosphorus Concentration

Through modulation of synthetic parameters including temperature, time, and OAm concentration, it was determined that Cu_3_P formation proceeded through a metallic copper intermediate that underwent phosphidation in the presence of sufficient OAm (15–20 mmol) at temperatures at or above 300 °C (Scheme 1). During modification of the reaction parameters, the phosphorus concentration was kept constant as the precursor, [Cu(H)(PPh_3_)]_6_, was the only source of phosphorus. Our previous report on the solution synthesis of metal phosphide NPs using single-source precursors indicated that increasing the P:M ratio through addition of excess PPh_3_ facilitated phosphidation.^28^ In a previous report of Cu_3_P NP synthesis, increasing the P:Cu ratio changed the mechanism of formation from phosphidation of a Cu intermediate to direct growth of Cu_3_P.^24^ To explore the effect of P:Cu ratio on Cu_3_P formation from [Cu(H)(PPh_3_)]_6_, the same reaction procedure was used, as previously discussed, but with the addition of 2 to 4 equiv of PPh_3_.

At 320 °C, the addition of 2 equiv of PPh_3_ resulted in Cu_3_P after 15 and 30 min reaction times (Figure S9) with average size distributions from TEM analysis of 8.6 ± 2.3 nm and 10.6 ± 2.3 nm, respectively (Figure S10). To differentiate between Cu_3_P synthesized with and without PPh_3_, P–Cu_3_P will be used to denote the NPs generated with added phosphorus. In a comparison of Cu_3_P NPs synthesized after heating for 15 min at 320 °C with and without PPh_3_, the addition of 2 equiv of PPh_3_ did not result in particle growth. Both conditions generated NPs with similar average size distributions of 8.7 ± 1.6 nm for Cu_3_P (Figure S1a) compared to 8.6 ± 2.3 nm for P–Cu_3_P. Elemental analysis revealed increased phosphorus concentrations at higher P:Cu ratios. The NPs synthesized with and without 2 equiv PPh_3_ contained 27 mol % P and 25 mol % P, respectively. The elemental analysis only provides the overall composition of Cu_3-x_P, and the P-concentration could contain contributions from unincorporated precursor decomposition products or surface-bound PPh_3_. Additionally, Cu vacancies, which will lead to a higher P-content than expected, are common for Cu_3-x_P, and the plasmonic behavior of Cu_3-x_P observed here and in previous work has been attributed to the presence of Cu vacancies (Figure S2).

To assess if Cu_3_P forms directly rather than through a metallic Cu NP intermediate at lower temperatures with increased P:Cu ratio, 4 equiv of PPh_3_ were added to the reaction and the mixture was held at 300 °C for 1–2 h (Figure S11). After 1 h, Cu_3_P was the major product; however, Cu was also observed, indicating that even in the presence of excess phosphine, Cu_3_P formation still proceeded through a Cu intermediate under these reaction conditions. After 2 h at 300 °C, XRD analysis indicated the formation of phase-pure Cu_3_P; however, the resultant product was a mixture of polydisperse aggregates analyzed by TEM (Figure S11b) and nondispersible material. Although Cu_3_P was accessed at the lower temperature of 300 °C with higher P:Cu ratios, the particles displayed significant aggregation and limited dispersibility. Poor dispersibility also resulted from reaction times greater than 1 h under most of the conditions utilized here. Understanding the influence of reaction parameters on phase-pure Cu_3_P formation, homogeneous particle morphology, and particle dispersibility, led to the selection of conditions of 30 and 15 min holds at 250 and 320 °C, respectively, in the presence of 15 mmol OAm with or without 2 equiv of PPh_3_ to generate Cu_3_P NPs for electrocatalytic studies.

### Electrocatalytic CO_2_ Reduction

FTIR spectroscopy of as-synthesized Cu_3_P particles (Figure S12a) revealed the distinct bands in the range of 3000–2800 cm^–1^ that can be assigned to C–H stretches that indicate the presence of residual organic species (e.g., OAm). Surface ligands, such as oleylamine, have been utilized to direct and improve CO_2_ reduction selectivity;^39^ however, previous electrochemical analysis on metal phosphide NPs have indicated that the presence of surface ligands reduces the electrochemically accessible surface area, thus inhibiting the catalytic activity.^40,41^ For electrochemical analysis, electrodes were prepared by drop-casting as-synthesized Cu_3_P NPs suspended in CHCl_3_ onto carbon paper (CP) to form OAm–Cu_3_P/CP. In order to evaluate the impact of surface bound oleylamine on the electrochemical performance, the OAm–Cu_3_P/CP was reduced at 450 °C in the presence of H_2_, a method that has been established to remove organic surface ligands from metal phosphide NPs.^28,42^ Retention of the Cu_3_P hexagonal phase following thermal treatment at 450 °C in the presence of H_2_ was confirmed by XRD analysis (Figure S13). Following thermal reduction, the reduced Cu_3_P-modified CP (R–Cu_3_P/CP) was passivated with 1% O_2_ to protect the surface from rapid oxidation upon air exposure.

**Figure 3:**
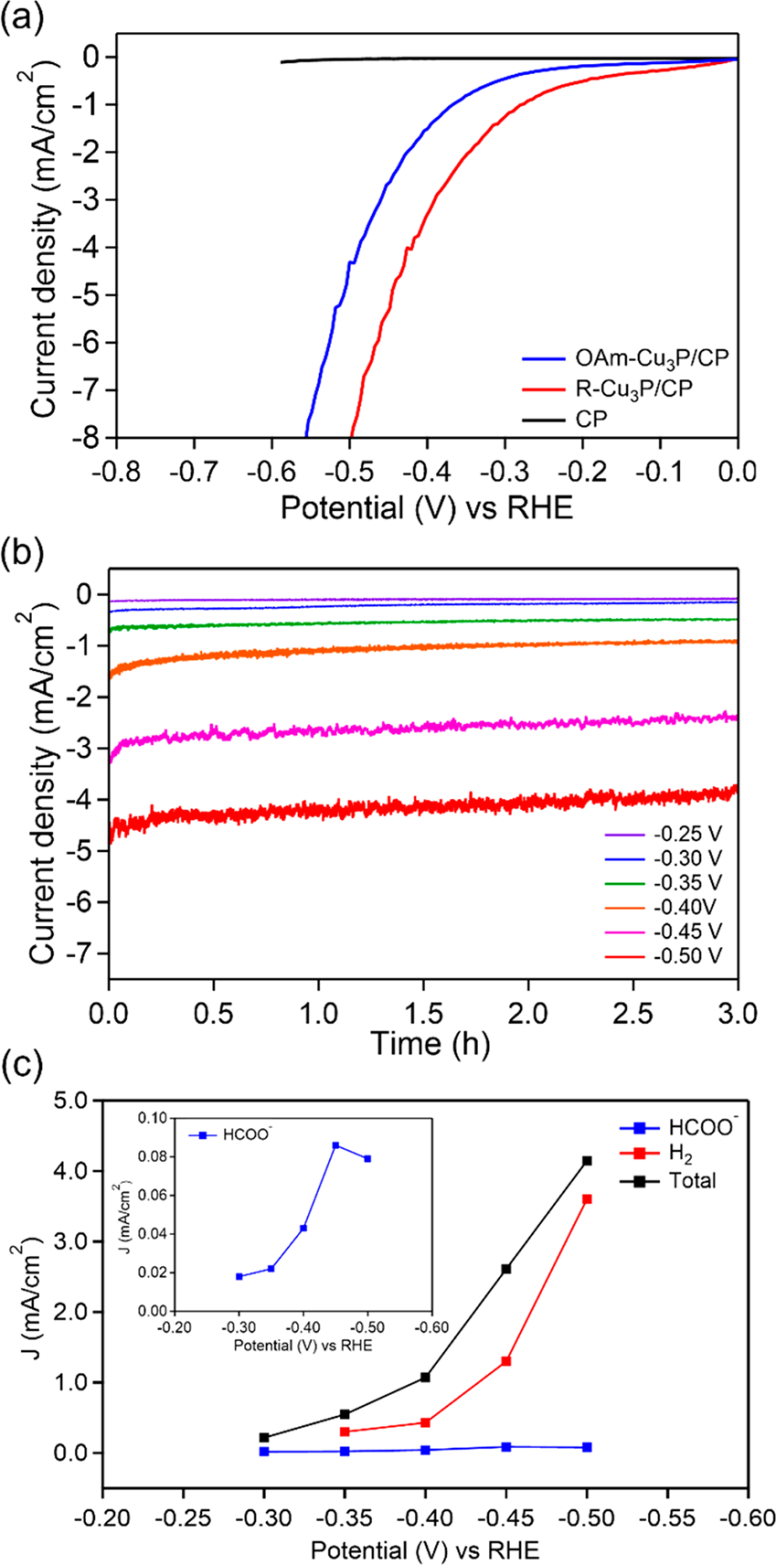
(a) Polarization curves of OAm–Cu_3_P/CP, R–Cu_3_P/CP, and carbon paper in CO_2_-saturated 0.1 M KHCO_3_ at a scan rate of 50 mV/s. (b) Chronoamperometry measurements of R–Cu_3_P/CP at different applied potentials (−0.25 to −0.50 V versus RHE) in CO_2_-saturated 0.1 M KHCO_3_. (c) Partial current densities for HCOO– (inset) and H_2_ for R–Cu_3_P/CP in CO_2_-saturated 0.1 M KHCO_3_.

The Cu_3_P/CP electrodes were electrochemically investigated in CO_2_-saturated 0.1 M KHCO_3_ aqueous electrolyte. To evaluate the influence of surface bound OAm on electrocatalytic CO_2_ reduction activity, electrochemical analysis was performed on both OAm–Cu_3_P/CP and R–Cu_3_P/CP. Evaluation of the capacitance current at the open circuit potential, which is directly related to the electrochemically accessible surface area, revealed increased capacitance for R–Cu_3_P/CP (Figure S14). Cyclic voltammograms indicated that R–Cu_3_P/CP had a more positive catalytic onset than OAm–Cu_3_P/CP (Figure 3a). The increased surface area and more positive onset potential following thermal reduction to remove surface ligands has been previously observed for metal phosphide NPs.^11,43^

The large current increase between −0.30 and −0.40 V versus RHE in the cyclic voltammograms is primarily attributed to electrocatalytic H_2_ evolution (Figure 3a). Electrolysis with OAm–Cu_3_P/CP and R–Cu_3_P/CP was performed for 3 h, at potentials ranging from −0.25 to −0.50 V versus RHE, to quantify gaseous and liquid products (Figure 3b, S15). At all potentials analyzed, H_2_ was the only gaseous product as well as the major product observed. At potentials more negative than −0.45 V versus RHE, H_2_ was generated with FE > 80%. Because of the dominance of H_2_ evolution at applied potentials more negative than −0.45 V, subsequent electrolysis experiments focused on potentials between −0.25 to −0.45 V versus RHE to assess if CO_2_ reduction could occur at potentials where the kinetics of H_2_ generation are diminished.

Electrolysis performed between −0.25 to −0.45 V (Figure 3b, S15) revealed production of formate as the only C-containing product with maximum FE of 8% and 6% reached at −0.30 V and −0.35 V for R–Cu_3_P/CP and OAm–Cu_3_P/CP, respectively (Tables 1, S1). Utilization of unmodified CP as an electrode for CO_2_ reduction under the same conditions resulted in minimal current generation and no formate production. The FE for H_2_ did not exceed 50% within the analyzed potential window and the rest of the current is tentatively attributed to charging of the interface, a nonfaradaic process,^44,45^ and reduction of the catalyst, the necessity of which is discussed in more detail below. Faradaic efficiencies closer to unity were obtained at applied potentials more negative than −0.45 V versus RHE, where the kinetics of H_2_ evolution dominated and formate production was diminished. Analysis of the partial current densities (Figure 3c) reveal formate production accounts for a minimal amount of the generated current during electrolysis especially at more negative potentials where the FE for H_2_ reaches unity. Nickel phosphide electrocatalysts have exhibited analogous CO_2_ reduction behavior, where CO_2_ reduction occurred at less negative applied potentials, and H_2_ production occurred at more negative potentials.^16^ Although CO_2_ reduction has been observed within a narrow potential window over nickel phosphide, silver phosphide, and copper phosphide catalysts, H_2_ evolution, which is thermodynamically and kinetically favored, is never fully suppressed. The exclusive reduction of CO_2_ to formate demonstrated here has also been observed over sulfur-modified copper. Sulfur-modified copper developed by Pérez-Ramírez et al. generated formate with FE of 80% at −0.80 V versus RHE in CO_2_-saturated 0.1 M KHCO_3_.^46^ The inclusion of sulfur modulated the adsorption of key intermediates thus switching the selectivity from the C–C coupled products typically produced over copper to formate.^46−51^ Following this concept, we propose a similar affect here, where the presence of P in the Cu_3_P/CP electrodes changes the intermediates versus metallic Cu, leading to formate production rather than C–C coupling.

**Table 1:** Faradaic Efficiency (FE) of Formate Production for R–Cu_3_P/CP in CO_2_-Saturated 0.1 M KHCO_3_

Potential (V) versus RHE	Formate FE (%)
–0.25	0.0
–0.30	8.0
–0.35	4.0
–0.40	4.0
–0.45	3.3
–0.50	1.9

The effect of increasing the potassium bicarbonate concentration from 0.1 to 0.5 M on the efficiency of formate generation was investigated for R–Cu_3_P/CP. As has been reported previously,^52^ the onset of catalysis was shifted to less negative potentials and larger current densities were generated as the electrolyte conductivity increased due to the higher bicarbonate concentration (Figure S16). Formate production began at −0.20 V versus RHE in 0.5 M KHCO_3_ in comparison to −0.30 V versus RHE in 0.1 M KHCO_3_; however, the maximum FE remained at 8% (Table S3). The reduction in overpotential for formate production in 0.5 M KHCO_3_ also corresponded to a reduction in overpotential for H_2_ production, resulting in increased FE for H_2_ at less negative applied potentials. The impact of phosphorus on the CO_2_ reduction activity was also explored. Cu_3_P NPs synthesized in the presence of 2 equiv of PPh_3_ are denoted as follows: OAm–P–Cu_3_P (as-synthesized) and R–P–Cu_3_P (reduced). OAm–P–Cu_3_P and R–P–Cu_3_P electrochemically reduced CO_2_ to formate at comparable FE as the Cu_3_P NPs synthesized without PPh_3_ (Figure S17).

**Figure 4:**
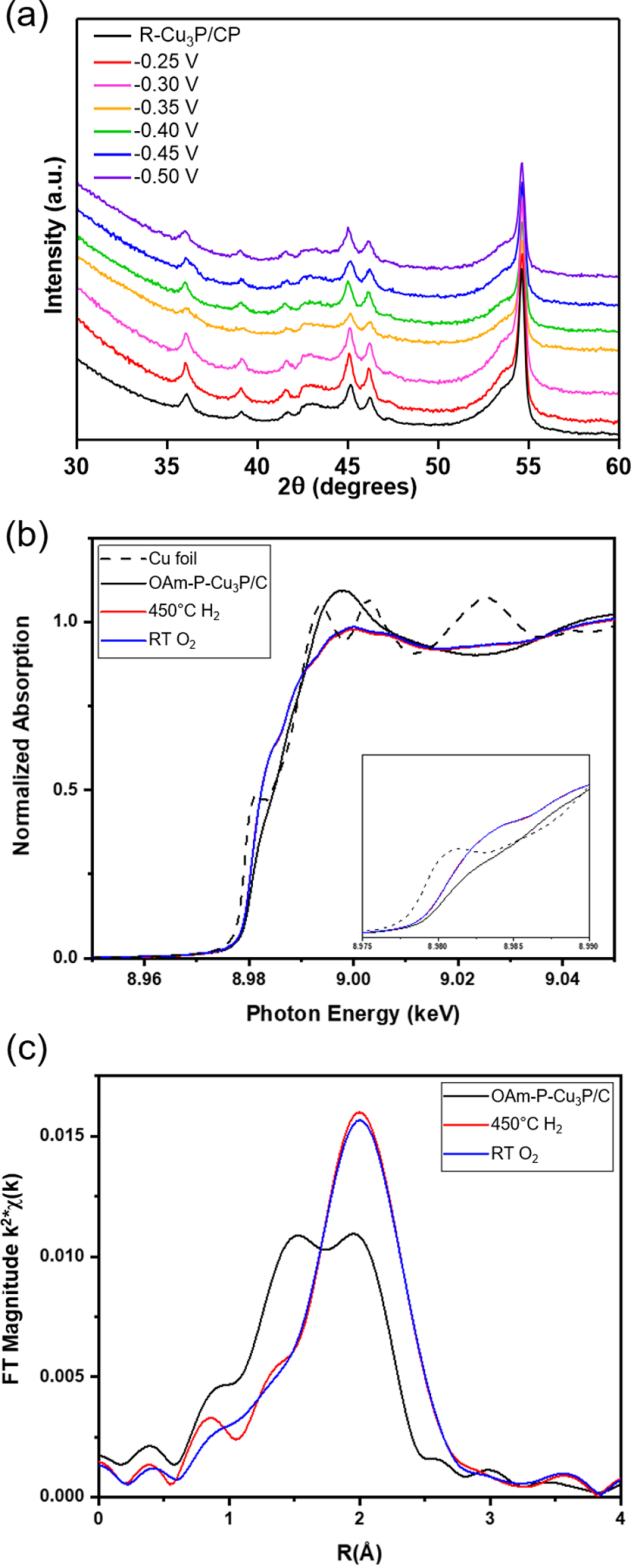
(a) XRD patterns of R–Cu_3_P/CP following 3 h electrolysis at potentials ranging from −0.25 to −0.50 V versus RHE in CO_2_-saturated 0.1 M KHCO_3_. (b) Normalized Cu K edge XANES for P-Cu_3_P/C before and after reduction at 450 °C in H_2_ and subsequent room temperature passivation in O_2_. (c) k^2^-weighted Cu K edge EXAFS of P-Cu_3_P/C.

Previous reports have identified that NPs commonly undergo transformations during electrocatalysis;^53^ therefore, it was necessary to evaluate the stability of Cu_3_P NPs during electroreduction in the aqueous conditions utilized here. For OAm–Cu_3_P/CP, a gradual increase in current density over time was observed (Figure S15) for electrolysis experiments performed from −0.25 to −0.50 V versus RHE in CO_2_-saturated 0.1 M KHCO_3_. To assess if the current instability was related to the surface OAm ligands, OAm–Cu_3_P NPs were drop-cast on FTO electrodes and FTIR spectroscopy was performed on the electrodes before and after 3 h electrolysis experiments at −0.40 V and −0.50 V versus RHE. Analysis of the FTIR spectra (Figure S12b) indicated that the C–H stretches associated with OAm persisted. However, the utilization of the transparent FTO electrode allowed for visual observation of electrode fouling (green discoloration) upon air exposure following the electrolysis experiments. The OAm–Cu_3_P/CP electrode was collected following 3 h electrolysis at −0.45 V versus RHE, and XRD analysis of the electrode was performed in air. The XRD pattern (Figure S18) showed diminished peaks associated with Cu_3_P revealing the electrochemical instability of this material. The peaks at 36° and 42.5° indicate the formation of copper oxide upon air exposure of the electrochemically polarized OAm–Cu_3_P/CP, which is consistent with the observed electrode discoloration. Therefore, the increase in current density observed during electrolysis of OAm–Cu_3_P/CP is due to the reductive transformation of the catalyst. The origin of the reduction-induced structural evolution of OAm–Cu_3_P is further discussed below. In contrast to the OAm–Cu_3_P/CP electrodes, which demonstrated an increase in current density over time, electrolysis with R–Cu_3_P/CP revealed a reduction in the generated current over 3 h (Figure 3b) and 6 h (Figure S19a) and a lower FE for formate after 6 h. The reduction in current and FE could be attributed to deactivation of R–Cu_3_P/CP over time. XRD of the R–Cu_3_P/CP electrodes performed following 3 and 6 h of electrolysis showed retention of the bulk structure with no evidence of crystalline copper oxides (Figure 4a, S19b). However, this analysis does not preclude the presence of other deactivation pathways such as transformation of the catalyst morphology and particle size or formation of amorphous degradation species. The impact of phosphorus on the electrochemical stability was also investigated. The XRD patterns of OAm–P–Cu_3_P/CP prepared from Cu_3_P NPs synthesized with 2 equiv of PPh_3_, following 3 h of electrolysis at −0.45 V versus RHE (Figure S18), also did not show crystalline copper oxide formation.

X-ray absorption spectroscopy (XAS) was performed to determine if differences in the geometric and electronic properties of the OAm–Cu_3_P, R–Cu_3_P, P–Cu_3_P NPs could explain the improved electrochemical stability for R–Cu_3_P and P–Cu_3_P. XAS was performed at the Cu K edge on carbon black-supported Cu_3_P NPs (Cu_3_P/C). The crystal structure of Cu_3_P contains four distinct Cu sites with different local arrangements of atoms, and XAS analysis provides an average of the Cu environments in the structure. Cu_3_P was analyzed in the following states: as-synthesized, thermally reduced at 450 °C in the presence of H_2_, and subsequently passivated with 1% O_2._ The instability observed electrochemically for OAm–Cu_3_P extended to the XAS experiments. OAm–Cu_3_P/C consistently degraded despite attempts to minimize air exposure during preparation and collection of the XAS data. The accelerated degradation could be attributed to the higher surface area carbon (Vulcan XC 72R) used for XAS analysis in comparison to the carbon paper employed for the electrochemistry experiments. Due to the inability to limit degradation of OAm–Cu_3_P and the similar electrocatalytic performance, only the OAm–P–Cu_3_P NPs were utilized for XAS experiments. Figure 4b shows the Cu K edge X-ray absorption near-edge structure (XANES) spectra of Cu_3_P NPs and Cu foil. The Cu_3_P NPs had a higher XANES energy (8.9805 keV) compared to the Cu foil (8.979 keV). The white line intensity of the as-synthesized Cu_3_P was higher than the Cu foil followed by dampened extended X-ray absorption fine structure (EXAFS) oscillations due to scattering from a lighter element (i.e., O versus Cu), likely due to the presence of small amounts of oxidized Cu. The K edge XANES represents an electron transition of the 1s to 3p electron and is sensitive to small changes in oxidation state. Following thermal reduction in H_2_, the white line intensity decreased relative to the as-synthesized Cu_3_P and the shape of the XANES spectrum broadened slightly. These changes correspond to a decreased energy of the unfilled states of Cu and can be due to both the loss of Cu oxides and the incorporation of P. The XANES energy increased slightly to 8.9806 keV for the H_2_ treated Cu_3_P NPs, likely because Cu sites bear a partial positive charge due to the electron density shift from the metal to the more electronegative phosphorus sites.^13^ No change in the XANES spectrum was observed following treatment of the thermally reduced Cu_3_P with 1% O_2_, indicating retention of the metallic character after surface passivation.

The EXAFS spectra were fit to determine the local coordination of Cu (Figure 4c, Figure S20). Because XAS is a bulk-averaged technique, the EXAFS fits are determined for an average Cu atom. In a pure phase Cu_3_P structure, there are four distinct Cu sites that contain an average Cu–P coordination number of 2.7 at 2.37 Å and average Cu–Cu coordination number of 3 at 2.68 Å (CN_Cu–P_/CN_Cu–Cu_ = 0.9). The OAm–P–Cu_3_P NPs were fit with Cu–O, in addition to Cu–P and Cu–Cu scattering pairs. The optimized EXAFS fit contained 1.6 Cu–O bonds at 1.92 Å, in addition to 1.5 Cu–P and 1.2 Cu–Cu bonds at 2.34 and 2.65 Å, respectively. The addition of P to form Cu_3_P elongates the Cu–Cu bond (2.65 Å) compared to what is expected in Cu NPs (2.51 Å).^54^ Here, the total coordination number was 4.3. This suggests that approximately 37% (1.6/4.3) of the nanoparticle was oxidized prior to reduction, though Cu_3_P was present. However, the absence of Cu-oxide reflections in the XRD pattern suggests the oxide species present are amorphous, perhaps on the nanoparticle surface, as opposed to crystalline, bulk copper oxide. After reduction, the Cu–O bonds were lost to the increased coordination of Cu–Cu and Cu–P with only Cu–Cu and Cu–P scattering pairs observed. The results suggest that prior to reduction, the Cu_3_P NPs were partially oxidized. The R–P–Cu_3_P NPs contained approximately 2.5 Cu–P bonds at 2.34 Å and 2.0 Cu–Cu bonds at 2.65 Å. The CN_Cu–P_/CN_Cu–Cu_ = 1.3 is higher than the expected CN_Cu–P_/CN_Cu–Cu_ = 0.9 for the ideal Cu_3_P structure, thus R–P–Cu_3_P NPs may contain Cu vacancies and/or excess or residual phosphorus species, which could result in a P-rich material (Table S4).^55^ Additional characterization is needed to determine the exact chemistries (Cu vacancies versus excess P-species) that lead to the diminished Cu–Cu contributions. There is no change to the EXAFS data following passivation of the reduced Cu_3_P NPs with 1% O_2_. Although bulk Cu–O contributions were not observed after passivation, XAS is not an appropriate technique to evaluate surface oxidation and thus, due to the air-sensitive nature of Cu_3_P, the presence of surface oxides following exposure to 1% O_2_, while not directly observed, is likely.

The XAS results provide some insight into the influence of thermal reduction and addition of PPh_3_ to the synthetic procedure on the stability of Cu_3_P NPs. The inability to collect XAS data on OAm–Cu_3_P confirms the material is unstable prior to electrochemical testing and degradation is accelerated during electrolysis. In comparison to OAm–Cu_3_P, the addition of 2 equiv of PPh_3_ during the synthesis to generate P–Cu_3_P helps promote phosphidation and possibly generate more robust and oxidatively stable NPs. Without additional PPh_3_, OAm–Cu_3_P is unstable, and although we were unable to collect XAS directly on OAm–Cu_3_P, we suggest this instability is due to a greater number of Cu–O bonds in the structure of OAm–Cu_3_P than OAm–P–Cu_3_P. We hypothesize that the addition of PPh_3_ during the synthesis facilitates isolation of Cu_3_P NPs more closely resembling the ideal structure and with a reduced number of Cu–O scattering pairs. Therefore, the contribution of Cu–O to the Cu_3_P structure is a key determinant of stability.

The extensive Cu–O bonds in OAm–P–Cu_3_P, revealed by XAS analysis and that we hypothesize are present in OAm–Cu_3_P, may provide an unstable catalytic surface under the electrochemical reduction conditions utilized. It is established that copper oxide is quickly reduced to metallic copper during CO_2_ electroreduction in aqueous media.^56,57^ Although XRD of OAm–P–Cu_3_P/CP following electrolysis did not show the same crystalline copper oxide formation as OAm–Cu_3_P/CP, the presence of Cu–O bonds in OAm–P–Cu_3_P as evidenced by EXAFS necessitates more extensive investigation into the material’s stability during electroreduction. XAS of Cu_3_P NPs reduced in H_2_ at 450 °C revealed the loss of the Cu–O bonds and an increase in the number of Cu–Cu and Cu–P bonds (Figure 4c, Table S1). Reductive pretreatment removes the unstable Cu–O contributions thus providing a more stable material for electrocatalysis. *In-situ* characterization is being pursued to provide more detailed insight into the impact of surface structure (as-synthesized versus reduced) and phosphorus stoichiometry on the electrocatalytic stability and performance of Cu_3_P NPs.

In addition to understanding the electrochemical stability of Cu_3_P NPs to facilitate the design of copper phosphide catalysts with improved electrocatalytic CO_2_ reduction activity, identification of the mechanism of formate production over Cu_3_P is also important. Because Cu_3_P can produce formate at small overpotentials, albeit at low FE, direct reduction of CO_2_ through a one-electron transfer to generate the radical anion or via a proton-coupled electron transfer (PCET) to produce adsorbed formate on the electrocatalyst surface is an unlikely pathway.^58^ Rather, the mechanism could proceed via an electrohydrogenation process whereby a proton and electron transfer occur simultaneously, coupling a surface hydride with CO_2_ in solution to generate formate. This electrohydrogenation pathway for the generation of formate from CO_2_ has been proposed for nickel phosphide catalysts. Nickel phosphide catalysts have been shown to produce C_3_ and C_4_ oxygenates through generation of formate via electrohydrogenation of CO_2_.^16^ It should be noted that CO production was not observed for the previously reported nickel phosphide catalysts or the copper phosphide catalysts evaluated here. CO has been identified as the key CO_2_ reduction intermediate to generate C_2+_ products,^20,22^ however, the proposed mechanism of CO_2_ reduction to C_3_ and C_4_ oxygenates over nickel phosphides does not rely on the formation of CO to facilitate C–C bond coupling. The presence of both nickel and phosphorus surface sites is proposed to preferentially stabilize oxygen-bound intermediates (HCOO*) versus the traditional carbon-bound intermediates (COOH*) on metals during CO_2_ reduction, which facilitates the production of the unique oxygenated molecules, methylglyoxal and 2,3-furandiol. Rigorous experimental and computational exploration into the mechanism of formate production over Cu_3_P is necessary to determine the viability of the electrohydrogenation pathway. Such insights have the potential to guide the synthesis of Cu phosphide-based materials with tailored composition or structure to control selectivity and promote higher FE to formate, and possibly carbon-coupled products.

## Conclusions

A facile molecular precursor route has been established for the preparation of solid, phase-pure Cu_3_P NPs. The decomposition of [Cu(H)(PPh_3_)]_6_, a commercially available precursor, at high temperature in a hydrocarbon/amine solvent mixture results in the generation of Cu_3_P NPs in a single-pot reaction. Evaluation of the mechanism of Cu_3_P formation revealed that the reaction proceeds via a Cu intermediate that undergoes increased rates of phosphidation at higher temperatures, longer reaction times, and in the presence of excess PPh_3_. Cu_3_P NPs prepared via the molecular precursor route were evaluated as electrocatalysts for the reduction of CO_2_ in CO_2_-saturated KHCO_3_ aqueous solutions. The highly dispersible nature of the Cu_3_P NPs allowed for facile deposition of the NPs from organic solvent onto carbon paper electrodes. The electrochemical behavior was investigated for the as-synthesized Cu_3_P that retained OAm surface ligands and Cu_3_P that was reduced in 5% H_2_ to remove the surface ligands. Unstable current response and electrode fouling during electrolysis was observed for the as-synthesized Cu_3_P, highlighting the instability of the material under the electrocatalytic conditions tested. The electrochemical stability of as-synthesized Cu_3_P NPs was improved with increasing phosphorus content. In contrast, the reduced Cu_3_P was markedly more stable than the as-synthesized material, and further electrochemical investigation revealed the production of formate beginning at −0.30 V versus RHE in CO_2_-saturated 0.1 M KHCO_3_ with a maximum FE of 8%.

Metal phosphides have recently been demonstrated to be a promising platform for electrochemical CO_2_ reduction, and this application of Cu_3_P NPs for the reduction of CO_2_ to formate builds on this pioneering work. Specifically, the utilization of nickel phosphides for the conversion of CO_2_ to C_3_ and C_4_ oxygenates has prompted a reevaluation of the application of this highly tunable and synthetically versatile materials class in the field of CO_2_ reduction. Continued investigations into the mechanism of formate production and the transformation of the catalyst surface during electrolysis are underway to compare the reaction pathways over Cu phosphides and Ni phosphides, and to determine the factors that are vital for facilitating CO_2_ conversion, such as phosphorus stoichiometry. These insights can assist in the design of advanced catalyst architectures, and the molecular precursor method we have developed for a wide-array of metal phosphide NPs provides a unique and advantageous pathway for the preparation of well-defined and highly controlled NPs to meet these designs. The utilization of the solution-based, molecular precursor route to highly processable Cu_3_P provides opportunities for tailoring the composition, morphology, and surface chemistry of Cu_3_P to achieve higher CO_2_ conversion efficiencies and potentially access more reduced and economically valuable oxygenates.

## Supporting Information Available

TEM images of Cu and Cu_3_P NPs, XRD patterns of Cu and Cu_3_P NPs formed by modulating reaction parameters, XRD patterns of Cu_3_P before and after electrolysis, Faradaic efficiency data, chronoamperometry data for OAm–Cu_3_P/CP, chronoamperometry data for R–Cu_3_P/CP in 0.5 M KHCO_3_, FTIR of Cu_3_P NPs before and after electrolysis, and capacitance data (PDF)

## Abbreviations

NPs: nanoparticles
CP: carbon paper
RHE: reversible hydrogen electrode
FE: Faradaic efficiency
NMR: nuclear magnetic resonance spectroscopy
HPLC: high-performance liquid chromatography
TEM: transmission electron microscopy
FTIR: Fourier transform infrared spectroscopy
XAS: X-ray absorption spectroscopy
XANES: X-ray absorption near edge structure
EXAFS: extended X-ray absorption fine structure
PXRD: powder X-ray diffraction.
