# Supplementary material for: Electrocatalytic CO2 Reduction over Cu3P Nanoparticles Generated via a Molecular Precursor Route
Source: ACS Appl Energy Mater. 2020 Oct 27;3(11):10435–46. doi: 10.1021/acsaem.0c01360 (PMC10905424; doi:10.1021/acsaem.0c01360)
Supplement: Supplementary file 1 — ae0c01360_si_001.pdf [file ae0c01360_si_001.pdf]

Supporting Information

Electrocatalytic CO_2 Reduction over Cu_3P Nanoparticles Generated via a Molecular Precursor Route

Courtney A. Downes[1], Nicole J. Libretto [2], Anne E. Harman-Ware[3], Renee M. Happs[3], Daniel A. Ruddy[1], Frederick G. Baddour[1], Jack R. Ferrell III[1], Susan E. Habas[1]*, and Joshua A. Schaidle[1]*

[1]Catalytic Carbon Transformation and Scale-Up Center, National Renewable Energy Laboratory, 15013 Denver West Parkway, Golden, CO 80401 (USA)

[2]Davidson School of Chemical Engineering, Purdue University, West Lafayette, IN 47907 (USA)

[3]Renewable Resources and Enabling Sciences Center, National Renewable Energy Laboratory, 15013 Denver West Parkway, Golden, CO 80401 (USA)

*Emails: susan.habas@nrel.gov, joshua.schaidle@nrel.gov

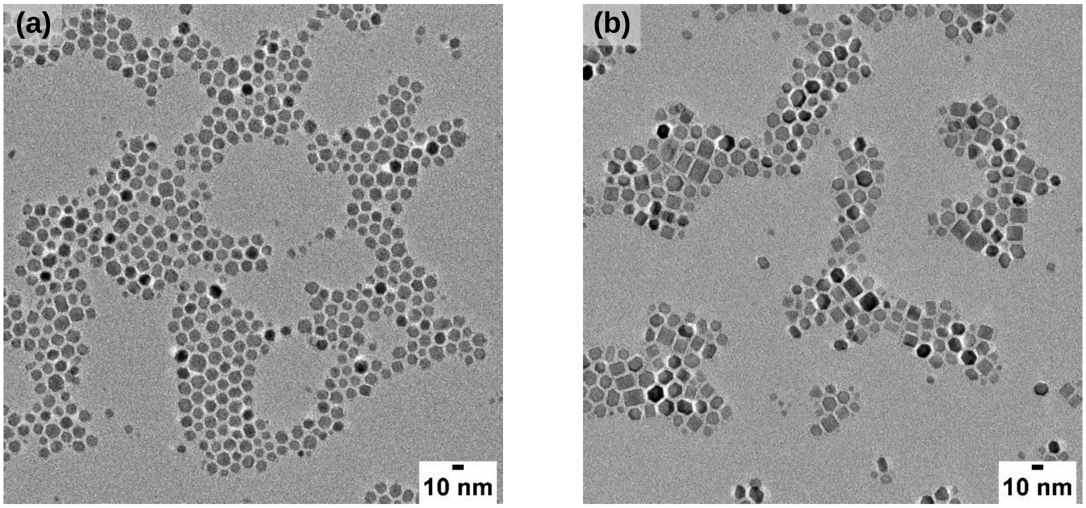

Figure S1. TEM images of Cu_3P synthesized with 15 mmol OAm following a 30 min hold at 250 °C and then a (a) 15 min hold or (b) 30 min hold at 320 °C.

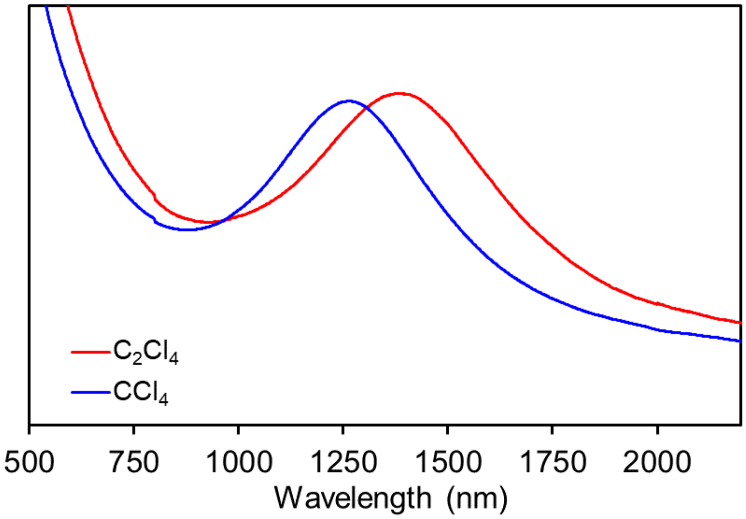

Figure S2. UV-Vis-NIR spectra of Cu_3P nanoparticles in 1,1,2,2-tetrachloroethane and carbon tetrachloride.

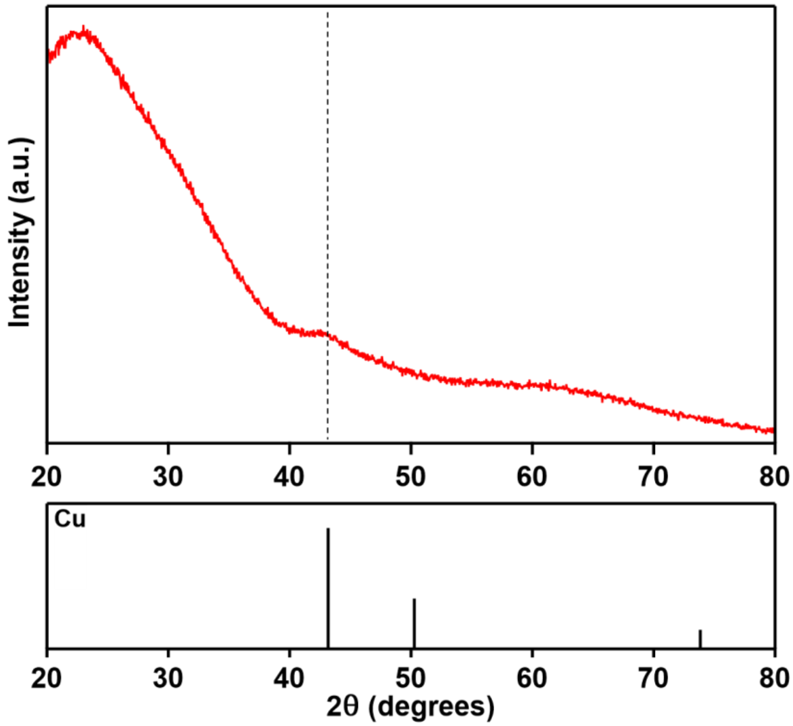

Figure S3. XRD pattern of reaction aliquot removed after 30 min at 250 °C with 15 mmol OAm. Reference pattern for Cu is shown below, and the dotted line on the experimental pattern indicates the highest intensity peak for Cu.

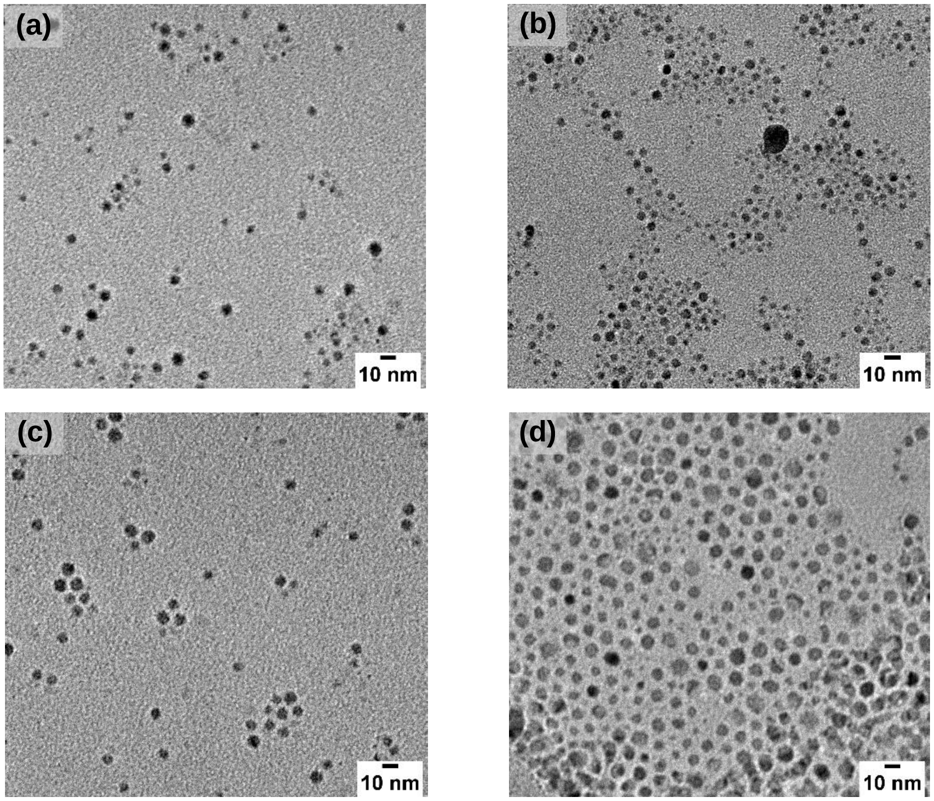

Figure S4. TEM images of aliquots removed (a) after 30 min at 250 °C, (b) immediately upon reaching 280 °C, and after (c) 5 min and (d) 1 h at 300 °C for reaction with 15 mmol OAm.

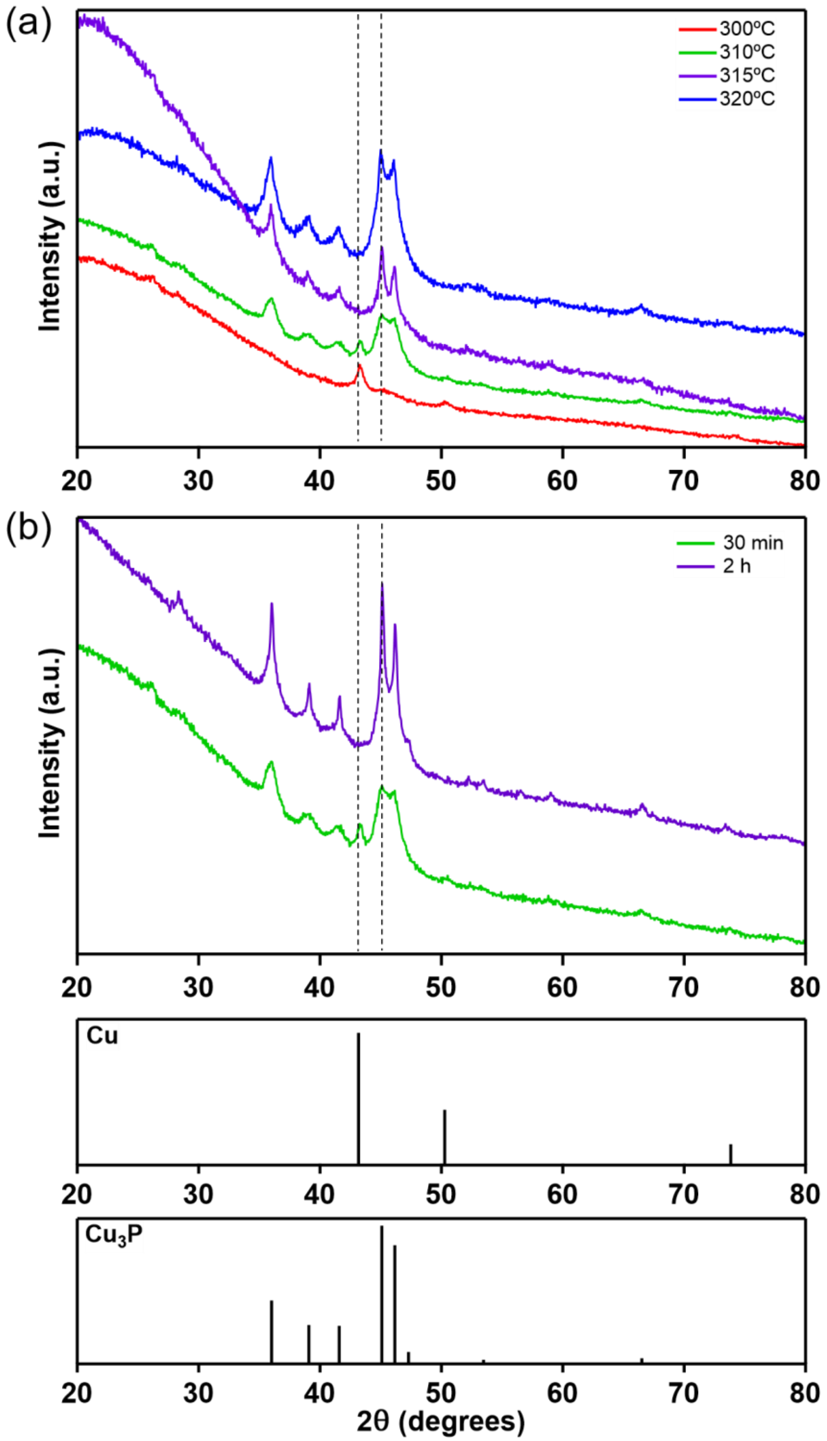

Figure S5. XRD patterns for NPs formed following (a) 30 min of heating at 300 °C, 310 °C, 315 °C, and 320 °C and (b) 30 min and 2 h of heating at 310 °C. Reactions were performed with 15 mmol OAm and included a 30 min temperature hold at 250 °C. Reference patterns for Cu and Cu_3P are shown below, and the dotted lines on the experimental patterns indicate the highest intensity peak for Cu and Cu_3P.

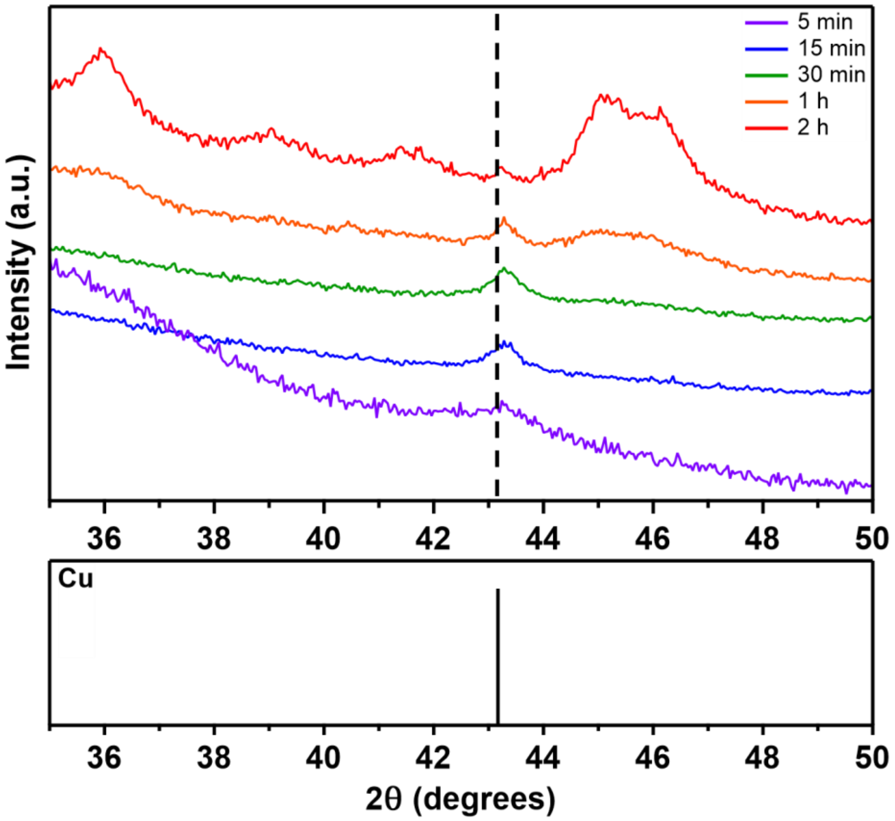

Figure S6. Expansion of the Cu region of the XRD patterns after 5 min, 15 min, 30 min, 1 h, and 2 h reaction at 300 °C with an oleylamine concentration of 15 mmol. Reference pattern for Cu is shown below, and the dotted line on the experimental patterns indicates the highest intensity peak for Cu.

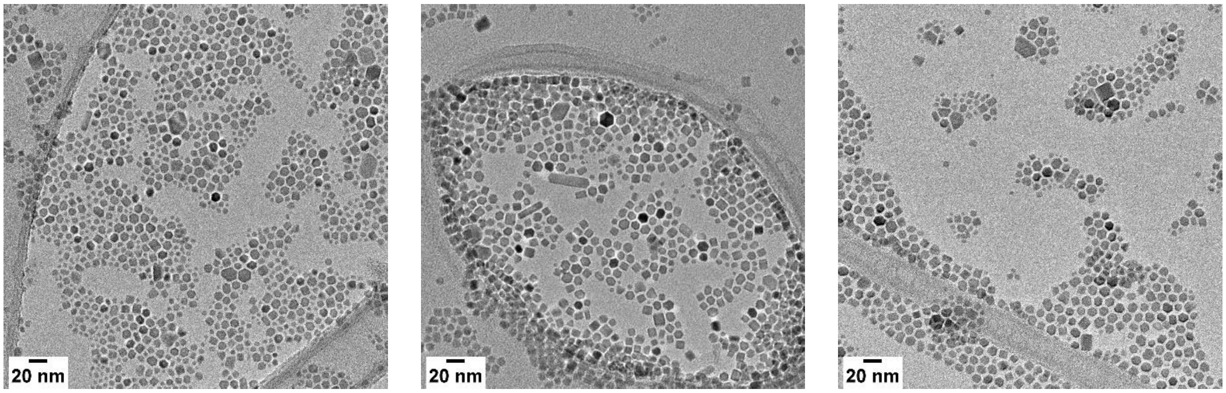

Figure S7. TEM images of Cu_3P synthesized with 20 mmol OAm following 30 min temperature hold at 250 °C and 15 min of heating at 320 °C.

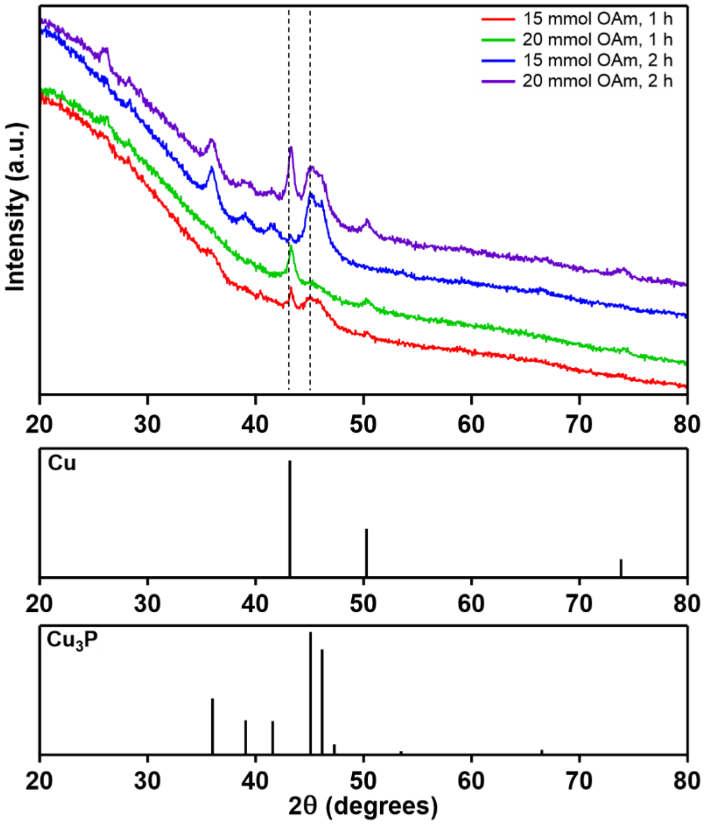

Figure S8. Comparison of XRD patterns after reaction at 300 °C with 15 mmol OAm and 20 mmol OAm for 1 h and 2 h. Reference patterns for Cu and Cu_3P are shown below, and the dotted lines on the experimental patterns indicate the highest intensity peak for Cu and Cu_3P.

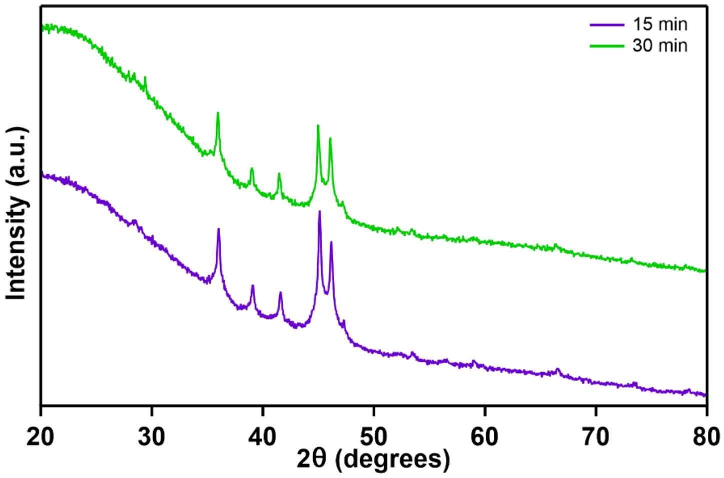

Figure S9. XRD patterns of Cu_3P NPs synthesized with 2 equivalents of PPh_3 and 15 mmol OAm and held at 320 °C for 15 and 30 min.

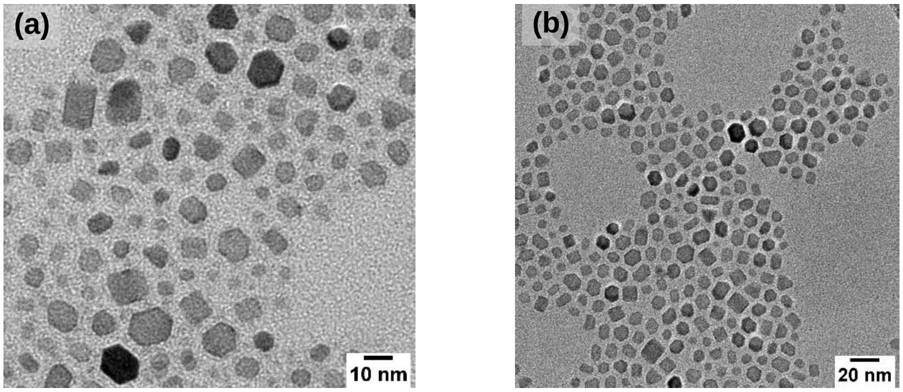

Figure S10. TEM images of Cu_3P synthesized with 15 mmol OAm and 2 equivalents of PPh_3 at 320 °C for (a) 15 min and (b) 30 min.

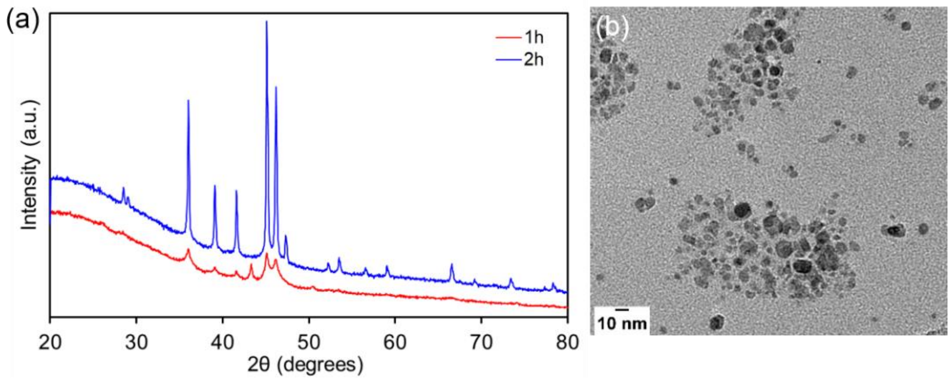

Figure S11. (a) XRD patterns of products formed with 4 equivalents of PPh_3 after 1 h and 2 h reaction at 300 °C. (b) TEM image of the reaction mixture following synthesis with 15 mmol OAm and 4 equivalents of PPh_3 at 300 °C for 2 h.

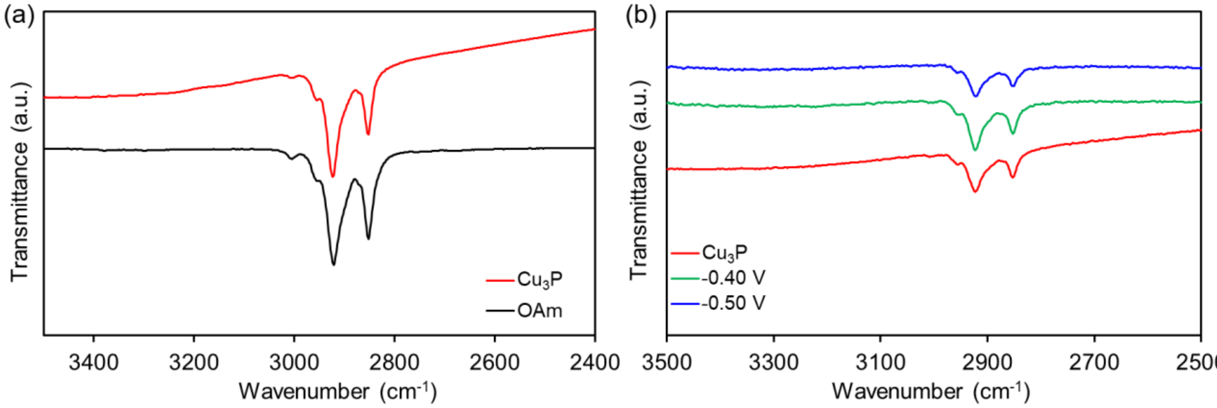

Figure S12. (a) FTIR spectra of as-synthesized Cu_3P NPs and neat OAm. (b) FTIR spectra of as-synthesized Cu_3P on FTO before electrolysis and following 3 h of electrolysis in CO_2-saturated 0.1 M $KHCO_3$ at -0.40 V and -0.50 V versus RHE.

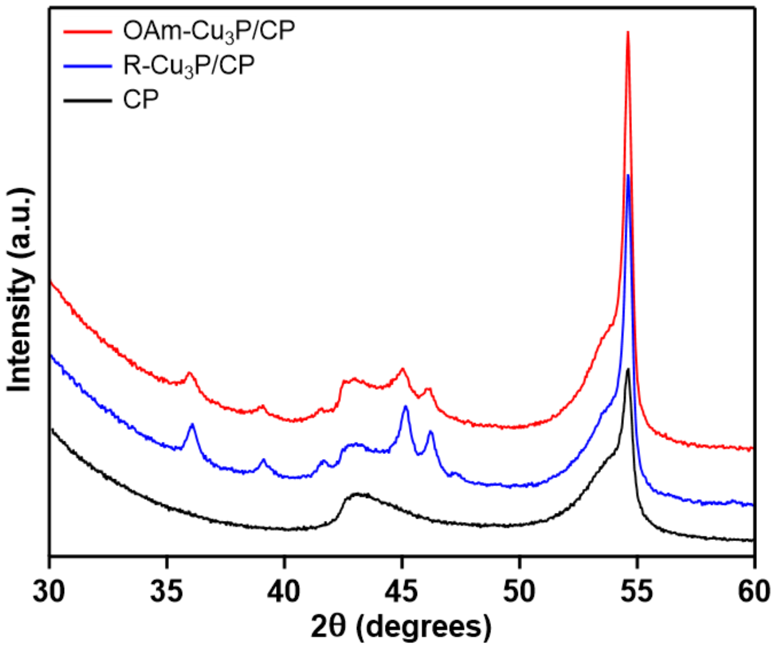

Figure S13. XRD patterns of OAm-Cu_3P/CP, R-Cu_3P/CP Cu_3P, and carbon paper.

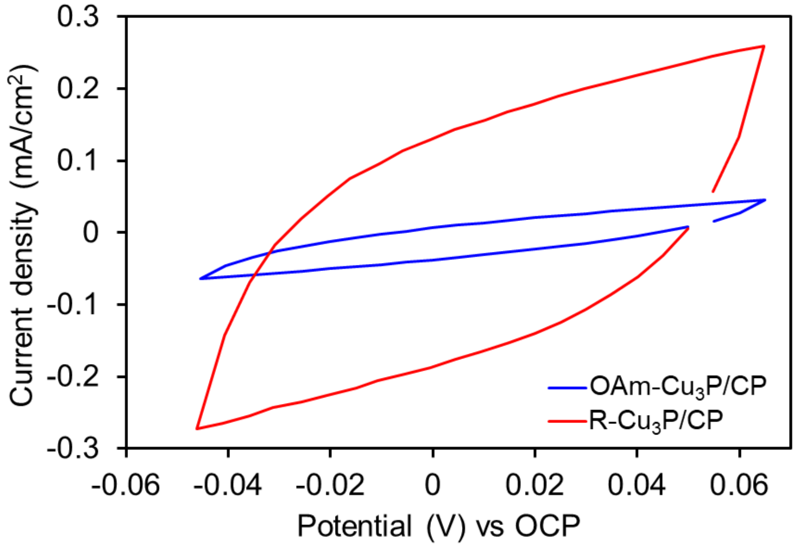

Figure S14. Capacitive current at the open circuit potential (OCP) of OAm-Cu_3P/CP and R-Cu_3P/C in CO_2-saturated 0.1 M $KHCO_3$ at a scan rate of 100 mV/s.

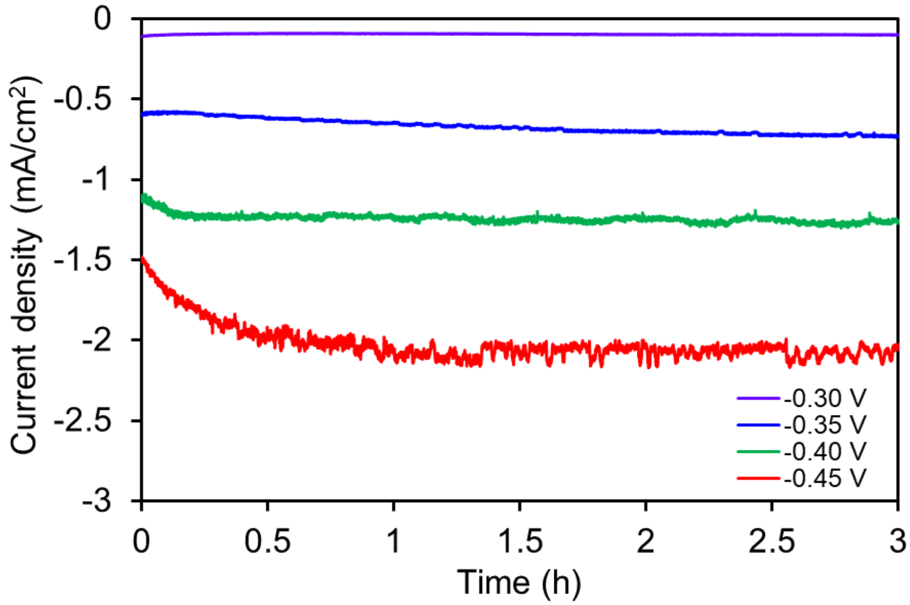

Figure S15. Chronoamperometry measurements at different potentials for OAm-Cu_3P/CP in CO_2-saturated 0.1 M $KHCO_3$.

Table S1. Faradaic efficiency for formate production, quantified by NMR, for OAm-Cu_3P/CP in CO_2-saturated 0.1 M $KHCO_3$.

Potential (V) versus RHE	Formate FE (%)
-0.35	6.0
-0.40	3.5
-0.45	4.0

Table S2. Faradaic efficiency for formate production, quantified by HPLC, for selected electrolysis experiments for OAm-Cu_3P/CP and R-Cu_3P/CP in CO_2-saturated 0.1 M and 0.5 M $KHCO_3$.

Catalyst	Electrolyte	Potential (V) versus RHE	Formate FE (%)
OAm-Cu_3P/CP	0.1 M $KHCO_3$	-0.35	6.8
OAm-Cu_3P/CP	0.1 M $KHCO_3$	-0.40	4.2
R-Cu_3P/CP	0.1 M $KHCO_3$	-0.35	3.6
R-Cu_3P/CP	0.1 M $KHCO_3$	-0.40	3.4
R-Cu_3P/CP	0.5 M $KHCO_3$	-0.20	7.2
R-Cu_3P/CP	0.5 M $KHCO_3$	-0.25	6.4

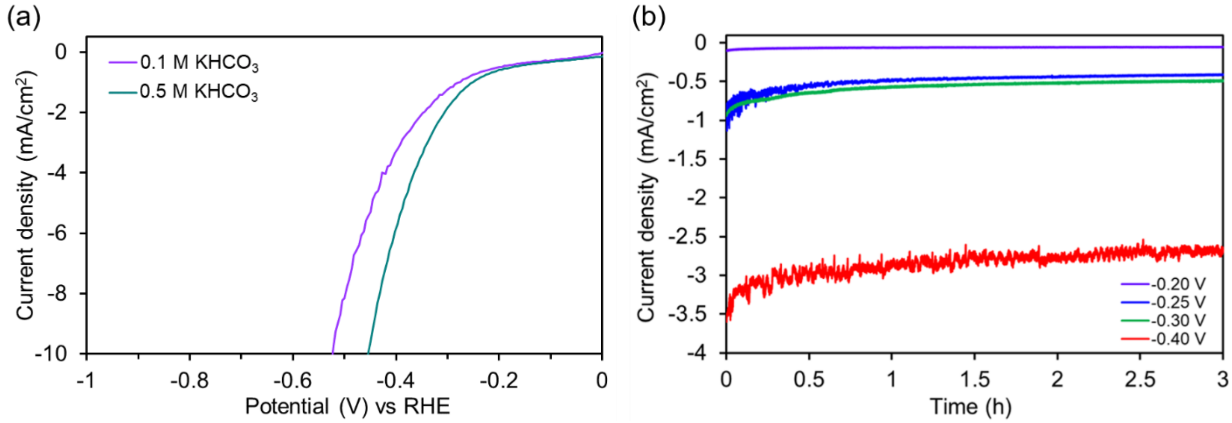

Figure S16. (a) Polarization curves for R-Cu_3P/CP in CO_2-saturated 0.1 M and 0.5 M $KHCO_3$ at a scan rate of 50 mV/s. (b) Chronoamperometry measurements at different potentials for annealed Cu_3P in CO_2-saturated 0.5 M $KHCO_3$.

Table S3. Faradaic efficiency of formate production, quantified by NMR, for R-Cu_3P/CP in CO_2-saturated 0.5 M $KHCO_3$.

Potential (V) versus RHE	Formate FE (%)
-0.20	8.0
-0.25	6.2
-0.30	4.2
-0.40	3.3

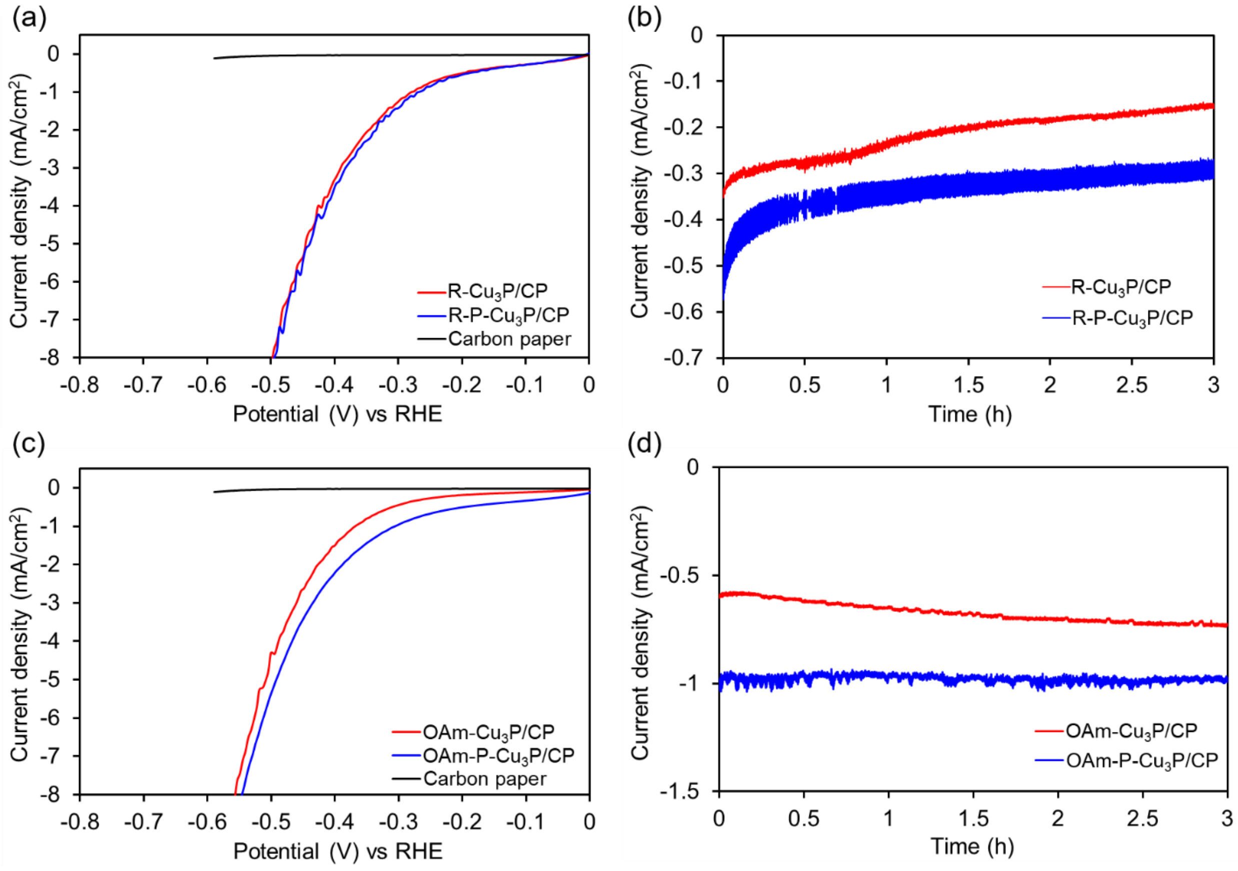

Figure S17. (a) Polarization curves for R-Cu_3P/CP and R-P-Cu_3P/CP in CO_2-saturated 0.1 M $KHCO_3$ at a scan rate of 50 mV/s. (b) Chronoamperometry measurements at -0.30 V versus RHE for R-Cu_3P/CP and R-P-Cu_3P/CP in CO_2-saturated 0.1 M $KHCO_3$. (c) Polarization curves for OAm-Cu_3P/CP and OAm-P-Cu_3P/CP in CO_2-saturated 0.1 M $KHCO_3$ at a scan rate of 50 mV/s. (d) Chronoamperometry measurements at -0.30 V versus RHE for OAm-Cu_3P/CP and OAm-P-Cu_3P/CP in CO_2-saturated 0.1 M $KHCO_3$.

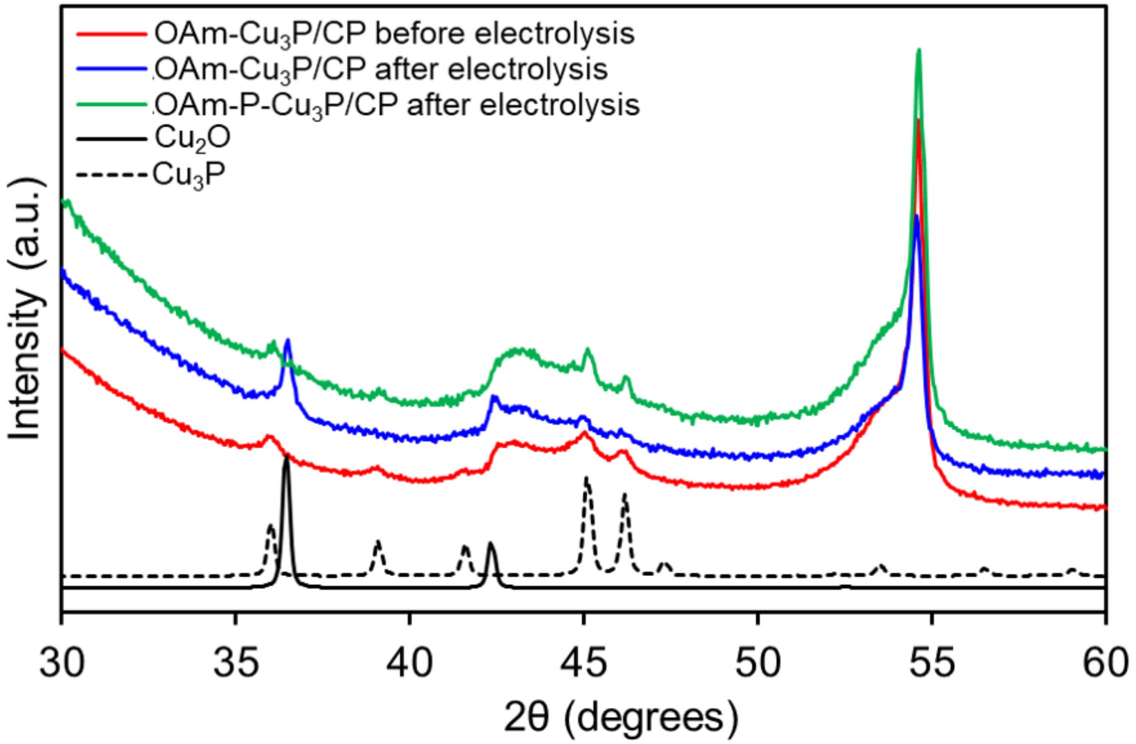

Figure S18. XRD patterns of OAm-Cu_3P/CP before electrolysis (red) and after 3 h electrolysis at -0.45 V versus RHE for Cu_3P synthesized without (blue) and with 2 equivalents of PPh_3 (green). XRD reference patterns for Cu_3P (solid black) and Cu_2O (dashed black) included.

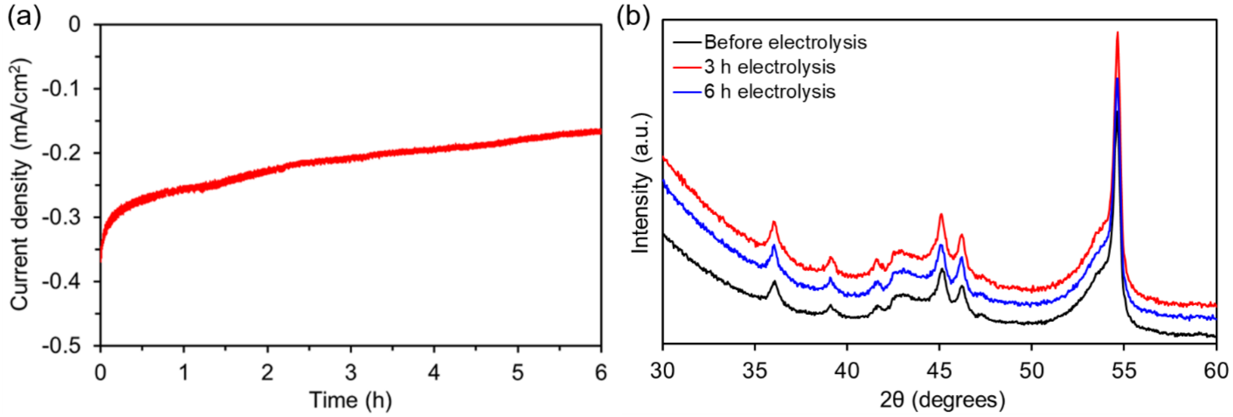

Figure S19. (a) Chronoamperometry measurement for R-Cu_3P/CP in CO_2-saturated 0.1 M $KHCO_3$ at -0.30 V versus RHE for 6 h. Faradaic efficiency for formate after 6 h was 2.9%. (b) XRD patterns of annealed Cu_3P on carbon paper before (black) and following 3 h (red) and 6 h electrolysis (blue) at -0.30 V versus RHE in CO_2-saturated 0.1 M $KHCO_3$.

Table S4. Fits of the k^2-weighted EXAFS for P-Cu_3P/C in He at room temperature, after reduction at 450 °C in 5% H_2/He, and after 1 h passivation in 1% O_2/He at 20 °C.

Treatment	XANES energy (keV)	Scattering Pair	CN	R (Å)	σ^2 (Å^2)	ΔE_0 (eV)
20 °C, He	8.9805	Cu-O	1.6	1.92	0.004	2.8
		Cu-P	1.5	2.34	0.004	
		Cu-Cu	1.2	2.65	0.004	
450 °C, 5% H_2/He	8.9806	Cu-P	2.5	2.34	0.004	1.4
		Cu-Cu	2.0	2.65	0.004	
20 °C, 1% O_2/He	8.9806	Cu-P	2.4	2.34	0.004	1.8
		Cu-Cu	2.1	2.65	0.004	

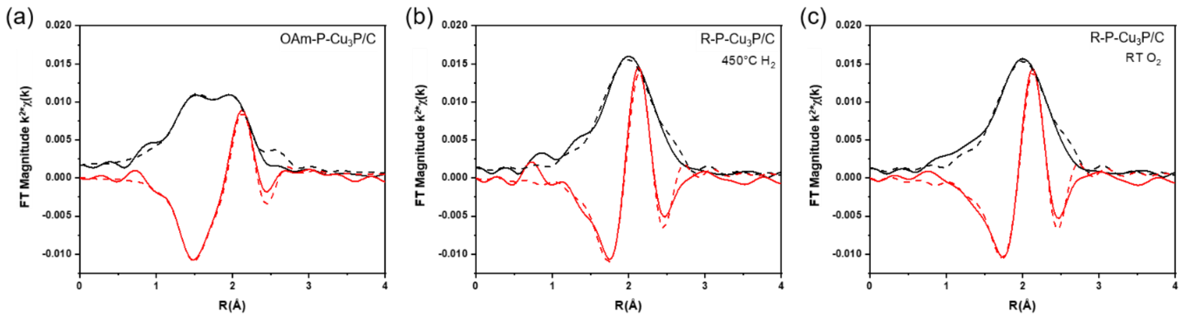

Figure S20. Quality of fits for P-Cu_3P/C (a) OAm-P-Cu_3P/C, (b) after reduction at 450 °C in 5% H_2/He, and (c) after passivation with 1% O_2/He at 20 °C, where the black traces represent the FT magnitudes and red represents the imaginary components with dashed lines for the fitted curves.
